# Curcumin Loaded PEGylated Nanoemulsions Designed for Maintained Antioxidant Effects and Improved Bioavailability: A Pilot Study on Rats

**DOI:** 10.3390/ijms22157991

**Published:** 2021-07-27

**Authors:** Jelena B. Đoković, Sanela M. Savić, Jelena R. Mitrović, Ines Nikolic, Bojan D. Marković, Danijela V. Randjelović, Jelena Antic-Stankovic, Dragana Božić, Nebojša D. Cekić, Vladimir Stevanović, Bojan Batinić, Jovana Aranđelović, Miroslav M. Savić, Snežana D. Savić

**Affiliations:** 1 Department of Pharmaceutical Technology and Cosmetology, Faculty of Pharmacy, University of Belgrade, Vojvode Stepe 450, 11221 Belgrade, Serbia; jelena.djokovic@pharmacy.bg.ac.rs (J.B.Đ.); jelena.mitrovic@pharmacy.bg.ac.rs (J.R.M.); ines.nikolic@pharmacy.bg.ac.rs (I.N.); 2 DCP Hemigal, Tekstilna 97, 16000 Leskovac, Serbia; saneladjordjevic87@gmail.com (S.M.S.); nesafarm@gmail.com (N.D.C.); 3 Department of Pharmaceutical Chemistry, Faculty of Pharmacy, University of Belgrade, Vojvode Stepe 450, 11221 Belgrade, Serbia; bojan.markovic@pharmacy.bg.ac.rs; 4 Department of Microelectronic Technologies, Institute of Chemistry, Technology and Metallurgy, University of Belgrade, Njegoševa 12, 11000 Belgrade, Serbia; danijela@nanosys.ihtm.bg.ac.rs; 5 Department of Microbiology, Faculty of Pharmacy, University of Belgrade, Vojvode Stepe 450, 11221 Belgrade, Serbia; jelena.stankovic@pharmacy.bg.ac.rs (J.A.-S.); dragana.bozic@pharmacy.bg.ac.rs (D.B.); 6 Department of Pharmaceutical Technology and Cosmetology, Faculty of Technology, University of Niš, Bulevar Oslobođenja 124, 16000 Leskovac, Serbia; 7 Department of Pharmacology, Faculty of Pharmacy, University of Belgrade, Vojvode Stepe 450, 11221 Belgrade, Serbia; vladimir.stevanovic@pharmacy.bg.ac.rs (V.S.); jovana.arandjelovic@pharmacy.bg.ac.rs (J.A.); miroslav.savic@pharmacy.bg.ac.rs (M.M.S.); 8 Department of Physiology, Faculty of Pharmacy, University of Belgrade, Vojvode Stepe 450, 11221 Belgrade, Serbia; bojan.batinic@pharmacy.bg.ac.rs

**Keywords:** curcumin, PEGylated nanoemulsions, long-term stability, experimental design

## Abstract

The current study describes the experimental design guided development of PEGylated nanoemulsions as parenteral delivery systems for curcumin, a powerful antioxidant, as well as the evaluation of their physicochemical characteristics and antioxidant activity during the two years of storage. Experimental design setup helped development of nanoemulsion templates with critical quality attributes in line with parenteral application route. Curcumin-loaded nanoemulsions showed mean droplet size about 105 nm, polydispersity index <0.15, zeta potential of −40 mV, and acceptable osmolality of about 550 mOsm/kg. After two years of storage at room temperature, all formulations remained stable. Moreover, antioxidant activity remained intact, as demonstrated by DPPH (IC50 values 0.078–0.075 mg/mL after two years) and FRAPS assays. In vitro release testing proved that PEGylated phospholipids slowed down the curcumin release from nanoemulsions. The nanoemulsion carrier has been proven safe by the MTT test conducted with MRC-5 cell line, and effective on LS cell line. Results from the pharmacokinetic pilot study implied the PEGylated nanoemulsions improved plasma residence of curcumin 20 min after intravenous administration, compared to the non-PEGylated nanoemulsion (two-fold higher) or curcumin solution (three-fold higher). Overall, conclusion suggests that developed PEGylated nanoemulsions present an acceptable delivery system for parenteral administration of curcumin, being effective in preserving its stability and antioxidant capacity at the level highly comparable to the initial findings.

## 1. Introduction

Curcumin is a dietary polyphenolic compound derived from turmeric (lat. *Curcuma longa*, fam. *Zingiberaceae*) with a long history of use as a traditional remedy in Chinese and Ayurvedic medicine [1,2]. It has also been the subject of scientific interest for over 50 years [3], given its anti-inflammatory, antioxidant, anticancerous, antiepileptic, antidepressant, immunomodulatory, neuroprotective, antiapoptotic, and antiproliferative effects [4]. Its therapeutic potential has been explored in the treatment of various conditions such as pulmonary problems, cancer, sepsis and Alzheimer’s disease [5,6,7,8].

However, wider therapeutic application of curcumin has been hindered by some of its physicochemical characteristics, such as poor aqueous solubility, degradation under alkaline conditions, heat and light sensitivity. Additionally, oral absorption of curcumin has been demonstrated to be low, making peroral administration route less effective for its delivery [2,9]. Therefore, the aim of our research was development of curcumin delivery system suitable for parenteral administration.

Nanoemulsions (NEs) stand out amongst other nanocarriers for being commercially available for both nutritive purpose (for over 50 years) and drug delivery systems. They have also found their place in food and cosmetic industries [10,11]. Moreover, they are biocompatible, biodegradable, and, depending on the preparation technique, relatively easy to prepare and handle. NEs possess uniform and small droplet size, high solubilization capacity for lipophilic drugs and good tolerability [12,13,14,15,16]. However, the use of intravenous nanoemulsions as drug carriers has been hindered by the rapid clearance of droplets from the blood by mononuclear phagocytic system (MPS). After intravenous administration, plasma opsonins adsorb to the surface of colloidal drug carriers, depending on carriers’ size and surface characteristics (hydrophilicity/hydrophobicity ratio), and subsequently facilitate phagocytosis by liver Kupffer cells, macrophages of spleen and bone marrow alongside with circulating monocytes and macrophages [14,15]. In order to achieve prolonged circulation time and reach target site, NE droplet size should be small enough, about 100–200 nm [16], and the droplets should have a hydrophilic surface. One of the approaches to increase the hydrophilicity of droplet surface is to introduce PEGylated phospholipid (PEG-PL) to the interface of the droplet—PEGylated NEs (PEG-NEs). Chains of PEG located on the droplet surface provide steric stabilization and reduce interactions with plasma proteins and cell surfaces [14].

Even though there are many publications regarding curcumin-based drug delivery systems, such as recently described pH sensitive nanomicelles or polysaccharides-based complex particles [17,18,19], among them some describing curcumin-loaded NEs [20,21,22,23,24], the majority of the research is focused on assessing the potential in vitro and in vivo effects of curcumin, and only a few are aimed towards NEs development [20,23]. To the best of our knowledge there are no publications concentrated on the developmental aspects of PEGylated curcumin-loaded NEs (C-PEG-NEs), which was of particular interest for our investigation.

The main focus of this research was using the design of experiments approach in the development of PEGylated nanoemulsions by exploiting formulation factors, as well as critical parameters of the preparation process, in order to obtain optimal placebo NEs. This approach, being used in the development of conventional nanoemulsions, had not been applied to PEGylated ones yet. Optimal conditions, chosen by the experimental design, were then used to prepare C-PEG-NEs, and their essential physicochemical properties, osmolality, viscosity, curcumin content, antioxidant potential and nano-droplet morphology were examined initially and followed during the two year storage period. In addition, in vitro release profiles for curcumin-loaded NEs were investigated in order to select the best candidates for further testing of curcumin antioxidant potential when it is encapsulated into nano-droplets as well as their impact on selected cell lines and in vivo pharmacokinetic behavior.

## 2. Results and Discussion

### 2.1. Preparation and Characterization of Nanoemulsions

One of the first considerations when developing NEs as drug delivery systems is the solubility and stability of the drug in oils, as it acts as a driving force for the selection of the oil phase [25]. Therefore, solubility of curcumin in soybean oil, alone and in combination with MCT was tested, considering their use in many formulations present on the market [26]. Nanoemulsions prepared only with soybean oil (containing long chain triglycerides—LCT) are characterized with bigger droplet size when comparing to the NEs based on the combination of soybean oil and MCT. Such a result could probably be explained by differences in viscosities between the oil phases. Lower viscosity of MCT and MCT–soybean oil mixtures vs. soybean oil alone means that oil droplets made of lower viscosity oils can be disrupted more efficiently during high pressure homogenization [27]. A fixed concentration of oil phase, being 20% *w*/*w*, was chosen due to higher triglyceride content in these emulsions compared to those containing 10% of the oil phase, lower phospholipid–triglyceride ratio and less interference with lipoprotein lipase activity [28]. Moreover, our aim was to choose the oil phase that would allow the incorporation of the highest concentration of curcumin, bearing in mind prospective in vivo pharmacokinetic studies on an animal model, and also a fact that there is no consensus on curcumin recommended doses range.

Solubility of curcumin in soybean oil alone, as well as in its mixtures with MCT was not satisfactory (<1.5 mg/mL), while the addition of lecithin resulted in slightly higher curcumin solubility (˂4 mg/mL). With the fixed concentration of the oil phase set at 20%, drug loading would be low with the use of oil mixture alone. Therefore, benzyl alcohol, a co-solvent mixable with oils, was used to maximize the incorporation of curcumin, and achieve the concentration of 0.75 mg/mL of curcumin in the NEs, given that the solubility of curcumin in benzyl alcohol was much higher compared to the oils and their mixtures (25 mg/mL). Finally, mixture of soybean oil and MCT in 1:4 ratio (*w*/*w*) was chosen as the oil phase, relaying additionally on our previous experience, in terms of providing NEs with small droplet size, good stability, and lower viscosity [16,29].

The composition of the aqueous phase has been dictated by the composition of the oil phase. Sodium oleate had to be added as an additional stabilizer; otherwise, NEs would fall apart within a week of preparing. Due to its addition, the pH of the formulations was about 8, which was unfavorable for curcumin’s stability [2,30], and consequently the pH of the NEs’ aqueous phase was set at around 7 by 0.1 M HCl.

Concordant with applied experimental design (Table 1), 25 unloaded nanoemulsion formulations were prepared using hot HPH and their physicochemical characterization was performed. Mean droplet size of all NEs was in the range of 106–171 nm, with PDI below 0.2 and ZP about −40 mV, suggesting that developed NEs could be suitable for parenteral use.

### 2.2. Experimental Design

Considering the impact of droplet size on the in vivo fate of nanoemulsions, their long term stability and success of aseptic filtration, as well as the significance of droplets size distribution for NEs’ safety profile [25,31,32], Z-ave and PDI were chosen as response variables in experimental design. This strategy was employed to study the evolution of droplet size and size distribution in relation to process parameters and formulation factors aiming to find the optimal combination of input variables yielding the NEs with smallest Z-ave and PDI. Specifically, this approach allowed to elucidate the impact of formulation factors (PEG-PLs type and concentration) and process parameters (HPH pressure and number of cycles) on the NEs critical properties (prospective critical quality attributes), and to point out at the same time to any interactions between the independent variables, which could not be seen by employing one factor at the time approach.

Following the applied D-optimal experimental design (Table 1), a total of 25 runs were proposed [15 (model points) + 5 (to estimate lack of fit) + 5 (replicates)] and then randomly performed. For the dependent variables, Z-ave and PDI, the effects corresponding to the investigated factors (PEG-PL type and concentration, HPH pressure and number of cycles) and their interactions were calculated (to see Z-ave and PDI for the NEs defined by experimental design, please check Table 1). Factors and interactions with insignificant (*p* > 0.05) influence on estimated responses were excluded from the generated models, except model terms required to support hierarchy, thus raising the reduced factorial models whose equations, in terms of coded factors, are given below in Equation (1) (Z-ave) and Equation (2) (PDI):

Z-ave = +127.88 + 1.43 A + 1.88 B − 12.37 C − 16.64 D(1) + 2.47 D(2) − 2.17 AC + 1.86 AD(1) + 0.38 AD(2) − 1.15 BC + 7.10 CD(1) + 0.58 CD(2)(1)

PDI = +0.14 + 0.009 B-0.016 C − 0.007 D(1) − 0.001 D(2) + 0.008 CD(1) + 0.006 CD(2)(2)

The ANOVA analysis showed the models generated for Z-Ave (model F value = 266.99) and PDI (model F value = 9.22) were significant (*p* < 0.05) with the non-significant lack of fit relative to the pure error. The proposed models had appropriate R^2^, adjusted R^2^, and adequate precision values (Z-Ave: 0.9956, 0.9919, 47.903; PDI: 0.7546, 0.6728, 10.195, respectively), indicating that two responses were well described by the proposed models and the models could be used to navigate the design space.

**Figure 1:**
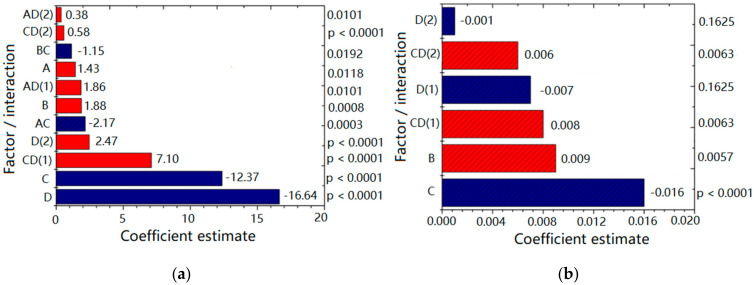
Plots for (**a**) mean droplet size—Z-Ave; (**b**) polydispersity index—PDI with the coefficient values and *p* values for the factors and their interactions. A: PEG-PL type; B: PEG-PL concentration; C: HPH pressure; D: number of HPH cycles.

Figure 1 depicts plots for the factors and interactions (with corresponding coefficient values and *p* values) influencing Z-ave (Figure 1a) and PDI (Figure 1b) of designed NEs. Higher coefficient value indicated a more pronounced effect on the investigated response, whereby a sign in front of coefficient indicated the direction of the model term influence; the negative sign pointed to the antagonistic effect of the adjoining factor. As can be seen from Figure 1a, the response Z-ave was significantly (*p* < 0.05) influenced by all the investigated factors in the following order: number of HPH cycles [D] > HPH pressure [C] > PEG-PL concentration [B] > PEG-PL type [A], with the process parameters (D and C) having a more pronounced effect compared to the formulation factors (A and B). By increasing the HPH pressure, Z-ave decreased (antagonistic effect). Higher homogenization pressure imposed bigger sheer force and turbulence on the processed formulation resulting in droplet size reduction [31]. Similarly, more HPH cycles usually led to Z-ave reduction, whereas the increase of PEG-PL concentration and the use of PEG5000-DPPE (compared to PEG2000-DSPE) led to increase of droplet size (synergistic effect). Furthermore, not only investigated factors alone, but also their interactions (HPH pressure/number of HPH cycles—CD; PEG-PL type/HPH pressure—AC; PEG—PL concentration/HPH pressure—BC; PEG-PL type/number of HPH cycles—AD) were shown to influence Z-ave at a significant level (*p* < 0.05), with CD being the most significant (Figure 1a).

On the other hand, PDI was significantly (*p* < 0.05) influenced by HPH pressure [C] and PEG-PL concentration [B], in the declining order (Figure 1b). Namely, PEG-PL concentration had a positive effect, meaning that the droplet size distribution was wider when 0.3% of PEG-PL was used compared to the 0.1%. On the contrary, the increase of HPH pressure produced narrower (desired) PDI (antagonistic effect). The number of HPH cycles [D] individually was not a significant factor for PDI, but it was kept in the model because the interaction between HPH pressure and the number of HPH cycles—CD has been shown as significant (Figure 1b).

**Figure 2:**
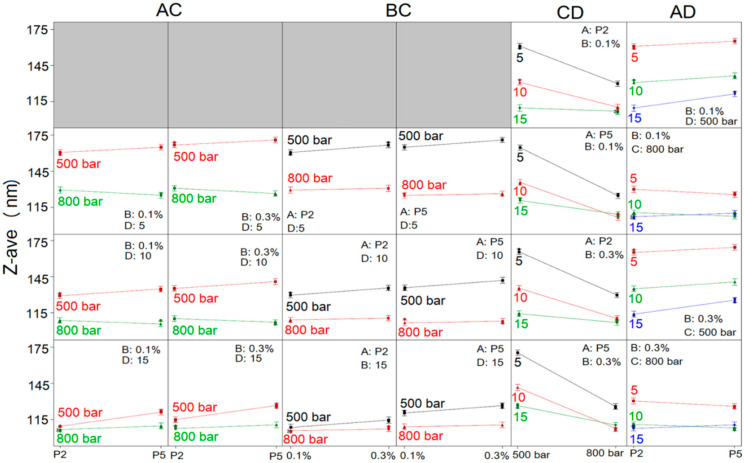
Interaction graphs for the mean droplet size (Z-Ave) of designed placebo nanoemulsions; P2-PEG2000-DSPE; P5-PEG5000-DPPE. AC—PEG-PL type/HPH pressure interaction; BC—PEG-PL concentration/HPH pressure interaction; CD—HPH pressure/number of HPH cycles interaction; AD—PEG-PL type/number of HPH cycles interaction.

**Figure 3:**
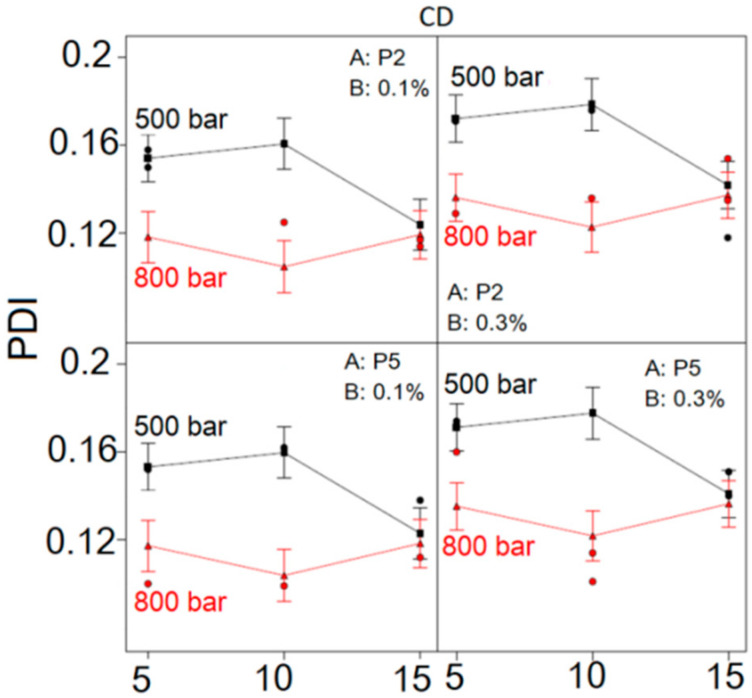
Interaction graphs for the polydispersity index (PDI) of designed placebo nanoemulsions; P2-PEG2000-DSPE; P5-PEG5000-DPPE.

To comprehend the full impact of investigated factors on NE droplet size and size distribution, the interactions between the factors were thoroughly studied, and the constructed interaction plots are shown in Figure 2 and Figure 3. It can be observed, through AC interaction plots (Figure 2), that the mean droplet size of NEs was lower when HPH pressure used for NE preparation was 800 bar compared to 500 bar, regardless of the type or concentration of PEG-PL or the number of homogenization cycles, whereby the difference in the Z-ave of NEs obtained under two different pressures was higher when fewer HPH cycles were employed (5 or 10 instead of 15 cycles). Furthermore, Z-ave of NEs prepared with PEG2000-DSPE at 500 bar was lower, as opposed to NEs formulated with PEG5000-DPPE, whether 0.1% or 0.3% of PEG-PL was used. On the contrary, when HPH was performed at 800 bar, Z-ave of NEs containing PEG5000-DPPE was slightly lower after 5 and 10 cycles of homogenization and slightly higher after 15 homogenization cycles, compared to NEs with PEG2000-DSPE, regardless of the PEG-PL concentration. From the plots describing the influence of BC interaction (Figure 2), it could be seen that the Z-ave of NEs produced with both PEG-PL types at HPH pressure of 500 bar, increased when the PEG-PL concentration increased from 0.1% to 0.3%, regardless of the employed number of homogenization cycles. When the HPH pressure of 800 bar was used, Z-ave of all obtained NEs was almost unchanged with increasing PEG-PL concentration.

Looking further at the CD interaction (Figure 2), it could be confirmed that with both PEG-PL types, at both concentrations, pressure of 800 bar delivered NEs with smaller droplets compared to 500 bar pressure, irrespective of the number of homogenization cycles; the decrease in Z-ave with increasing pressure was more pronounced when number of HPH cycles applied was 5 and 10, while the change in Z-ave was less noticeable in case of 15 homogenization cycles and especially when PEG2000-DSPE was used as PEG-PL. Moreover, the process factors’ combination of 800 bar/10 cycles yielded NEs with the smallest Z-ave in case of NEs formulated with PEG5000-DPPE, whereas in case of NEs based on PEG2000-DSPE no significant difference in droplet size was observed after 10 and 15 cycles of homogenization at 800 bar. In the AD interaction plots (Figure 2) it was observed that, regardless of the PEG-PL concentration, increase of homogenization cycles from 5 to 15, reduced the Z-ave of PEG2000-DSPE NEs; the decrease in Z-ave is more pronounced at 500 bar compared to 800 bar. The same scenario was observed with PEG5000-DPPE NEs produced at 500 bar—Z-ave decreased as the number of cycles increased. Slight deviation from this observation was seen when PEG5000-DPPE NEs were produced at 800 bar, where the smallest droplet size was obtained with 10 homogenization cycles.

Considering the influence of CD interaction on NE droplet size distribution (Figure 3), it could be observed that PDI was lower at HPH pressure of 800 bar, regardless of the number of HPH cycles, type and concentration of PEG-PL, compared to the PDI of formulations made at 500 bar. The difference in PDI between NEs produced at 500 and 800 bar was pronounced when the number of applied HPH cycles was 5 and 10, whereas with 15 homogenization cycles this difference became negligible. At the HPH pressure of 500 bar, the increase of HPH cycles from 5 to 10 led to increase of PDI, whereas further increase of HPH cycles from 10 to 15 caused the PDI of NEs to decrease. On the contrary, at HPH pressure of 800 bar, the initial decline of PDI between HPH cycles 5 and 10 was followed by an increase between cycles 10 and 15, irrespective of PEG-PL type and concentration. Likewise, the use of higher PEG-PL concentration led to increase in PDI, independent of PEG-PL type, HPH cycles and pressure. In general, the lowest PDI was observed at HPH pressure of 800 bar after 10 homogenization cycles, disregarding PEG-PL type or concentration.

Taking into consideration all the individual factors, as well as their interactions, numerical optimization process was used in order to choose the formulations with smallest Z-ave and PDI. In that vein, the program chose the top 10 formulations (out of 25 possibilities, including repeated runs, see Table 1) and assigned each one a desirability value. Higher desirability value indicated better accordance with set goals—minimized Z-ave and PDI (Table 2). According to obtained results, both PEG2000-DSPE and PEG5000-DPPE could be used in both proposed concentrations (0.1% and 0.3%), while HPH pressure of 800 bar and 10 homogenization cycles were chosen as optimal process parameters. These were the templates for curcumin loaded formulations.

To verify validity of the optimal parameters and calculated predicated responses, three of the proposed optimized nanoemulsion formulations (solutions 1, 2, and 5; Table 2) were selected. Three batches of each formulation were prepared, and obtained experimental data for Z-ave and PDI were compared with predicted ones. The observed experimental values from the confirmation experiment were within the calculated prediction interval, that is, in agreement with the predicted values (Supplementary Materials, Table S1), suggesting that the developed experimental design could be successfully used for evaluation and optimization of nanoemulsion formulation and manufacturing method, within the actual design space.

### 2.3. Curcumin-Loaded Nanoemulsions—Characterization and Stability Evaluation

Based on the results of experimental design, PEGylated nanoemulsions loaded with curcumin were prepared with the addition of 0.1%/0.3% of PEG2000-DSPE (CP21 and CP23, respectively) in oil phase or 0.1%/0.3% of PEG5000-DPPE (CP51 and CP53, respectively) in aqueous phase (Table 3), employing the homogenization at 800 bar for 10 cycles. The comprehensive evaluation of obtained nanoemulsions, together with the control non-PEGylated formulation (CNPEG), regarding their physicochemical properties, morphology and two-year stability during storage at room temperature, was performed. Upon preparation, all curcumin-loaded NEs were yellow in color and homogeneous in appearance. Average droplet size was 101–108 nm, with narrow distribution (PDI < 0.15), indicating suitability for parenteral application [33]. Absolute values of ZP were above 30 mV (Table 4), pH values between 6.76 and 6.89 and viscosity was in the range 23–46 mPa*s (Table 5). Additional LD measurement confirmed the absence of droplets bigger than 1 µm with d100 below 0.258 µm (Supplementary Materials, Figure S1). Osmolality values for the CNPEG, CP21, CP23, CP51 and CP53 were 556, 555, 531, 562 and 543 mOsm/kg, respectively. This would indicate good tolerability upon parenteral administration, as osmolality values higher than 600 mOSm/kg have been reported to cause shriveling up of red blood cells and significant pain, while osmolality values less than 150 mOsm/kg may cause hemolysis [34].

When comparing CNPEG to C-PEG-NEs (Table 4), a slight (less than 10 nm), but statistically significant increase in average droplet size was observed (*t*-test, *p* < 0.001). This could indicate that the maximal decrease in droplet size has already been achieved and further addition of stabilizers (PEG-PLs) cannot additionally reduce droplet size. On the contrary, due to the hydrophilic nature of the PEG chains covering the droplets surface, the hydrodynamic radius measured by DLS technique and expressed as Z-ave, was enlarged [14,35]. There were no statistically significant differences in Z-ave between placebo and curcumin-loaded formulations, except when 0.3% of PEG-PLs were used (*t*-test, *p* < 0.001, CP23 vs. P23; *p* < 0.01, CP53 vs. P53), where curcumin-loading caused decrease in average droplet size. This could indicate interactions between curcumin and stabilizing layer of nanoemulsions for CP23 and CP53 [36]. Absolute values of ZP about 40 mV indicated a good chance for the long term stability. The highest absolute value of zeta potential in curcumin-loaded NEs was observed in CNPEG, while the addition of PEG-PLs caused decrease in zeta potential values in C-PEG-NEs (Table 4). This could be explained by the fact that PEG chains provide a steric coverage of the droplet surface, thus reducing the zeta potential value compared to the CNPEG, which should not be taken as a sign of decreased stability [35]. In NEs stabilized with 0.1% of PEG2000-DSPE/PEG5000-DPPE, the addition of curcumin led to changes in ZP values, a decrease of absolute ZP values for CP21, and an increase for CP51 (*t*-test, *p* < 0.05), while the differences in ZP between other placebo and corresponding curcumin-loaded NEs were not statistically significant (Table 4).

**Figure 4:**
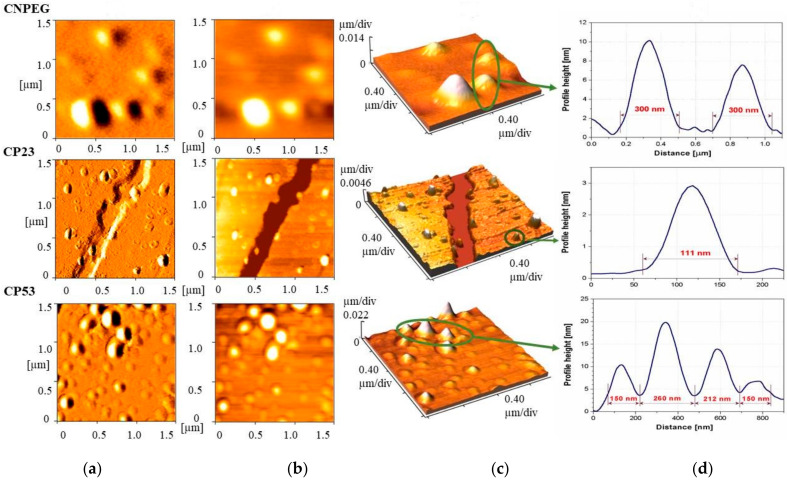
AFM images of curcumin loaded nanoemulsions—non PEGylated (CNPEG) and two PEGylated containing 0.3% of PEG2000-DSPE (CP23) or PEG5000DPPE (CP53): (**a**) error signal of 1.5 × 1.5 µm^2^ area of the sample; (**b**) 2D topography; (**c**) 3D topography; (**d**) height profiles of selected nanoemulsion droplets.

In order to provide additional information regarding droplet size, size distribution, morphology and potential aggregation of curcumin loaded samples, AFM analysis was performed. The AFM images depicting error signal, 2D and 3D topography from the selected samples—the non-PEGylated CNPEG and two PEGylated NEs—CP23 and CP53 are shown on Figure 4a, 4b and 4c, respectively. Height profiles for the selected droplets are presented in Figure 4d. AFM photomicrographs showed slightly larger droplet diameter of CNPEG NE than data from DLS and LD analyses suggested, possibly due to the drying process used for sample preparation in AFM that might cause the droplet merger. On the other hand, the photomicrographs of the PEGylated samples showed droplet sizes concordant with the DLS and LD measurements. It appeared that droplets of CP23 sample were more spherical in shape, while the droplets of CP53 were more elliptical. Additionally, it would appear that the droplets of CP23 were better defined compared to the other two samples, possibly indicating a more compact interlayer formed by PEG2000-DSPE compared to PEG5000-DPPE. However, such assumption should be further investigated by other techniques, such as electron paramagnetic resonance spectroscopy. No undissolved drug crystals were detected in any of the formulations.

Considering the pore diameter for aseptic filtration is 0.22 µm, droplet size should be below 200 nm to avoid the loss of droplets and incorporated drug. Droplet size (Z-ave) was measured prior to and upon the filtration to find out the impact of the sterilization process on droplet size and PDI and results are shown in Table 6. Although there were some differences in PDI observed between the formulations before and after filtration, additional LD measurements confirmed absence of larger droplets (Supplementary Materials, Figure S2), proving the aseptic filtration did not impact physicochemical characteristics of the formulation.

The stability of prepared curcumin-loaded NEs was followed for two years, which was particularly relevant considering the shelf life of some marketed formulations—such as Diazemuls^®^, 5 mg/mL emulsion for injection (Accord Healthcare Limited, Harrow, UK) or Diprivan^®^, 20 mg/mL emulsion for injection or infusion (Aspen Pharma Trading Limited, Dublin, Ireland) is two years, according to their summary of product characteristics (SmPC). Upon visual inspection there were no signs of NEs phase separation in the vials during the two-year storage period. Although the change in average droplet size (Table 4) was statistically significant for all curcumin-loaded NEs (*t*-test, *p* < 0.05), except for CP21, the slight increase in Z-ave (up to 10 nm for C-PEG-NEs and about 30 nm for CNPEG) still deemed them acceptable for parenteral application. Such findings may indicate an efficient stabilizing role of the PEG-PLs during the long-term storage period. After two years, PDI values remained below 0.25 (Table 4), indicating narrow droplet size distribution and the absence of coalescence of droplets. LD measurements, performed after one year of storage showed no significant change in droplet size (Supplementary Materials, Figure S1). More importantly, the absence of a population of larger droplets was confirmed. Some larger droplets could be observed under optical microscope at 1000× magnification (Supplementary Materials, Figure S3) after two years of storage, but this is somehow expected when preparing NEs with HPH technique and, taken together with results obtained from other characterization techniques, was not interpreted as a sign of instability. No signs of curcumin crystals were detected. Absolute ZP values of the curcumin-loaded NEs (Table 4), changed slightly during storage (*t*-test, *p* < 0.05), while still remaining above 30 mV.

After two years storage, pH values of investigated NEs dropped by approximately 1 unit, from about 7 to about 6 (Table 5). With the exception of CP53, the decrease of pH was more pronounced in non-PEGylated (CNPEG) compared to the PEGylated formulations, which was attributed to the fact that the PEG chains on the droplets surface may provide protection against the degradation of lecithin and the release of free fatty acids stabilizing pH in that way [36]. Viscosity of the curcumin-loaded formulations and their conductivity also decreased during storage (Table 5), which was not seen as a significant influence on the safety of nanoemulsions. After two years osmolality values for CNPEG, CP21, CP23, CP51 and CP53 were 538, 547, 539, 543 and 546 mOsm/kg, respectively, remaining in the same acceptable hypertonic range. As another stability indicator, concentration of curcumin in the formulations did not decrease during the two-year storage period (Supplementary Materials, Figure S4), additionally supporting the claim of stability for designed curcumin-NEs.

### 2.4. Antioxidant Assay

Due to the fact that most antioxidants are multifunctional, more than one type of assay should be performed in order to take into account the various mechanisms of antioxidant action [37]. DPPH assay is based on the reduction of DPPH free radical in the presence of a hydrogen donating antioxidant, resulting in the formation of the non-radical form of DPPH, which is manifested by the loss of violet color [38]. The degree of discoloration corresponds to the radical scavenging potential of the investigated antioxidant [39]. The assessment of curcumin, tocopherol and BHT through this assay showed that that the antioxidant potential of curcumin and tocopherol were similar (Supplementary Materials, Figure S5), with IC50 values of 0.1195 ± 0.0015 and 0.1023 ± 0.0045 mg/mL, respectively, coinciding with previous research results [23]. However, scavenging activity of BHT appeared to be much lower at the same concentrations (Figure S5, Supplementary Materials), potentially because BHT alone has shown less hydrogen donating ability compared to the tocopherol [40], which is the main mechanism of antioxidant action in this assay.

Antioxidant potential of curcumin-loaded nanoemulsions (CNPEG, CP21, CP51) assessed through DPPH assay indicated that curcumin maintained its antioxidant potential, as evidenced by the IC50 values obtained after testing freshly prepared formulations and the ones kept for two years at room temperature (Table 7), which is in line with the curcumin chemical stability in the formulations (Supplementary Materials, Figure S4). It can also be noted the scavenging activity of the selected formulations was higher compared to the curcumin alone (lower IC50 values), which could be explained by the presence of BHT in the formulations.

**Figure 5:**
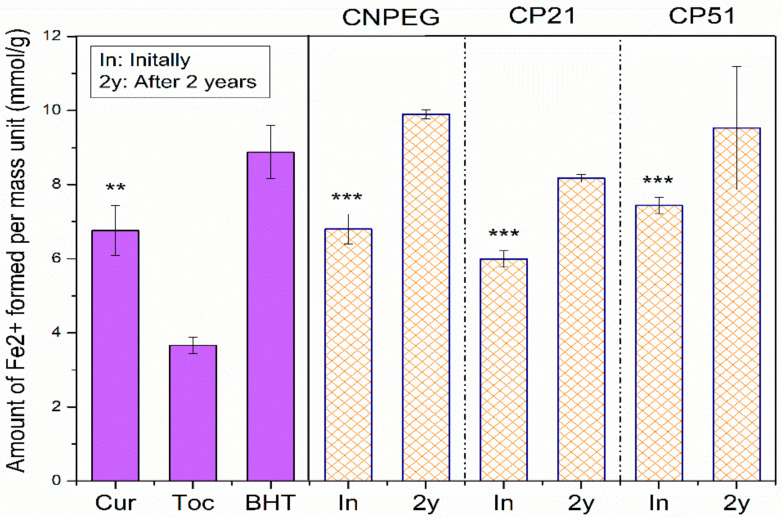
FRAP assay values for antioxidants and curcumin-loaded nanoemulsions: means ± sd (*n* = 3); ** and *** *p* < 0.01; *p* < 0.001, compared to tocopherol.

On the other hand, FRAP assay is based on the antioxidant induced reduction of ferric tripyridyltriazine complex to the ferrous tripyridyltriazine, at low pH [23]. Another point of distinction between these two assays lies in the fact that FRAP assay is performed in aqueous surroundings where the droplet structure is preserved. FRAP values for tocopherol were significantly lower compared to curcumin and nanoemulsion formulations (CNPEG, CP21 and CP51)—3.66 ± 0.22 mmol Fe^2+^/g versus 6.80 ± 0.40, 5.99 ± 0.22 and 7.44 ± 0.22 mmol Fe^2+^/g, respectively (*p* < 0.05, Student’s *t*-test), probably due to the phenolic structure of curcumin which is known to impact FRAP values [37]. On the other hand, there were differences between FRAP values of curcumin and its nanoemulsions (CNPEG, CP21, CP51), but these distinctions were deemed statistically insignificant (*p* = 0.937; *p* = 0.135 and *p* = 0.175 for curcumin versus CNPEG, CP21 and CP51, Student’s *t*-test). FRAP values of the formulations did not decrease after two years of storage, confirming the preservation of antioxidant potential during storage, with retention percentage values of 145.56%, 136.36% and 128.09% for CNPEG, CP21 and CP51, respectively, compared to the initial results (Figure 5), which was found acceptable compared to the values obtained in other FRAP assays assessing the antioxidant potential of different fruits during storage [41].

### 2.5. In Vitro Drug Release

**Figure 6:**
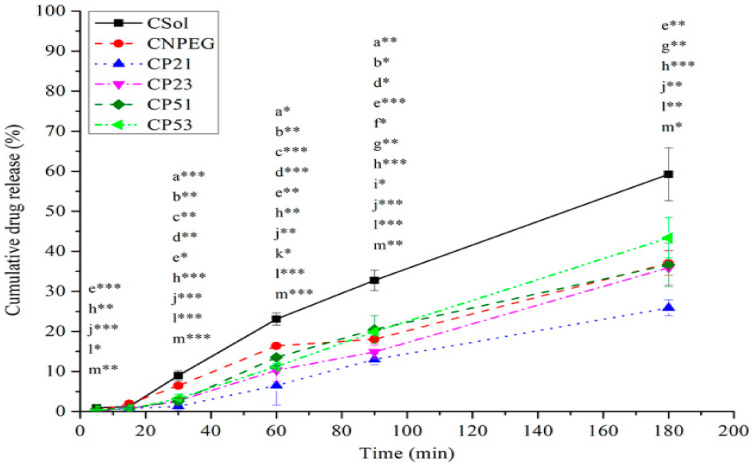
Cumulative % of curcumin released from loaded formulations (t = 37 °C). Values are displayed as means ± sd (*n* = 3); a* *p* < 0.05, a** *p* < 0.01, a*** *p* < 0.001, CNPEG vs. CP21; b* *p* < 0.05, b** *p* < 0.01, CNPEG vs. CP23; c** *p* < 0.01, c*** *p* < 0.001, CNPEG vs. CP51; d* *p* < 0.05, d** *p* < 0.01, d*** *p* < 0.001, CNPEG vs. CP53; e* *p* < 0.05, e** *p* < 0.01, e*** *p* < 0.001, CNPEG vs. CSol; f* *p* < 0.05, CP21 vs. CP51; g** *p* < 0.01, CP21 vs. CP53, h** *p* < 0.01, h*** *p* < 0.001, CP21 vs. CSol; i* *p* < 0.05, CP23 vs. CP53, j** *p* < 0.01, j*** *p* < 0.001, CP23 vs. CSol; k* *p* < 0.05, CP51 vs. CP53; l* *p* < 0.05, l** *p* < 0.01, l*** *p* < 0.001, CP51 vs. CSol; m* *p* < 0.05, m** *p* < 0.01, m*** *p* < 0.001, CP53 vs. CSol.

In vitro release of curcumin from the selected NEs was studied via reverse dialysis bag technique, which is considered to mimic in vivo situation for NEs and other colloidal systems upon their intravenous administration [42,43,44]. It could be observed from the release profiles shown in Figure 6 that after 60 min the highest fraction of curcumin was released from the non-PEGylated formulation (CNPEG—16.38% ± 0.23%, compared to 6.49% ± 4.93%, 10.28% ± 2.79%, 13.51% ± 0.48% and 11.20% ± 0.8% released from CP21, CP23, CP51 and CP53, respectively). This was expected, given that the PEG chains provide steric coating around the droplet, slowing down the release of curcumin from the PEGylated NE droplets [14]. Interestingly, after 90 min, the fraction of curcumin released from CP53 was bigger compared to CNPEG (19.92% ± 0.89% vs. 18.04% ± 0.17%, *p* < 0.05), but after 180 min there were no statistically significant differences in fractions of curcumin released from CNPEG and C-PEG-NEs. The only statistically significant difference in the fraction of released curcumin after 180 min was between CP21 and CP53 (25.88% ± 1.97% vs. 43.38% ± 4.99%, *p* < 0.05). It should be noted that the fraction of curcumin released from all of the formulations was significantly lower (*p* < 0.05) compared to the curcumin solution (CSol) at every time point, except after 15 min when there were no statistically significant differences in the fractions of curcumin released between any of the formulations (Figure 6). This could be expected, given that curcumin is encapsulated inside the nanoemulsions, and it has to, firstly be released from the oil droplets and then pass the dialysis bag membrane, whereas with CSol the only limiting step is the dialysis bag itself. The difference between the release of curcumin between the CSol and nanoemulsions provides a better insight as to the encapsulation efficacy of the carrier itself. Interestingly, the increase of the PEG2000-DSPE concentration (from 0.1% to 0.3%) did not further slowdown the release of curcumin from the nanoemulsions (CP21 and CP23, respectively). In fact, the release of curcumin was lower from the CP21 formulation compared to the CP23, while the similar trend did not apply to the CP51 and CP53 NEs, where there were no statistically significant differences between the fractions of curcumin released, except at 60 min time point (*t*-test, *p* < 0.05). Therefore, it could be noted that, while both types of PEG-PLs contributed to lower release of curcumin from the C-PEG-NEs compared to the CNPEG, PEG2000-DSPE had a bigger impact compared to the PEG5000-DPPE. The influence of the concentration of the PEG-PLs on the curcumin release was not that clear, given its low influence on the CP51 and CP53, and that an increase of PEG2000-DSPE concentration did not further lower the fraction of curcumin released, but had an opposite effect. Given the increase of PEGylated phospholipid concentration did not further slowdown the release of curcumin from the PEGylated formulations, the CP23 and CP53 formulations were excluded, leaving CP21 and CP51 as better candidates for further research.

In order to gain deeper insight into curcumin release kinetics from investigated NEs, the experimental data obtained from the release assay were fitted into different mathematical models—zero-order, first-order, Higuchi, Hixson–Crowell, Baker–Lonsdale, Korsmeyer–Peppas [45,46], and the parameters calculated are reported in Table S2, Supplementary Materials. Based on the highest values of the coefficient of determination (R^2^) and the adjusted coefficient of determination (R^2^ adjusted), and the lowest Akaike information criterion (AIC) [45,46], the best fitting of the experimental data was obtained with the Hixson–Crowell model for CNPEG and zero-order for the PEG-NEs (please see Table S2, Supplementary Materials). These models were chosen as optimal to interpret the release kinetics of curcumin from investigated nanoemulsions.

By analyzing the release rate constant of PEG-NEs (Supplementary Materials, Table S1, zero-order model), it could be noticed that the release rates of curcumin from CP21 and CP23 were significantly lower compared to the CP51 (*t*-test, *p* < 0.05, CP21 vs. CP51) and CP53 (*t*-test, *p* < 0.05, CP21 vs. CP53 and CP23 vs. CP53). This was probably due to the fact that DSPE provides a more rigid droplet interface compared to the DPPE [14], resulting in retarded release, which should be corroborated in further investigations. PEG-NEs are best described by zero-order kinetic model used to describe dosage forms that do not disaggregate, indicating that the nanoemulsion droplets remained intact during testing, thus providing controlled release [45,46]. On the other hand, the non-PEGylated formulation is best described by Hixson–Crowel model, suggesting that nanoemulsion droplets were destructed during the release testing, and the release rate was limited by the curcumin’s dissolution rate, but not by the diffusion through the stabilizing layer. This furthermore corroborates the stabilizing effects of PEG-PLs on nanoemulsions.

### 2.6. Cytotoxic Assay and Hemolysis

**Figure 7:**
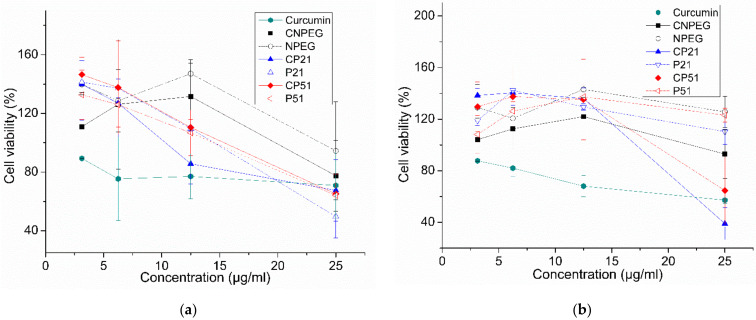
Cell viability (%) for investigated formulations evaluated for (**a**) MRC-5 cell line and (**b**) LS-174. All values are presented as mean ± sd (*n* = 3).

In vitro cell viability assay was conducted in order to estimate the biological impact of selected nanoemulsion formulations. Firstly, the effect of the formulations was assessed towards the MRC5 cell line in order to check their safety profile. It could be observed from Figure 7a that neither of the tested curcumin-loaded (CNPEG, CP21, CP51) nor their corresponding placebo formulations (NPEG, P21, P51) had an effect on this cell line in the investigated concentration range, indicating good safety profile. An increase of the formulation concentration in the cell medium led to slightly diminished effect on cell viability, which could probably be explained by the presence of benzyl alcohol in the formulations, which can act as preservative and has been demonstrated to have a concentration dependent cytotoxic effect [47].

Safety of the curcumin-loaded formulations was further corroborated with hemolysis test results that found them to be nonhemolytic, with the percentage of hemolysis 7.17 ± 0.73%, 2.65 ± 0.39% and 2.96 ± 3.44% for CNPEG, CP21 and CP51, respectively [48]. It should be noted that, even though all formulations were nonhemolytic, the non-PEGylated formulation (CNPEG) had more than two-fold increase in hemolytic activity compared to the PEGylated nanoemulsions (CP21 and CP51), probably due to the PEG chains hindering the interactions with erytrocytes. Additionally, this test was significant in confirming the safety of the formulations chosen for in vivo testing, given that it was performed using rat blood and undiluted formulations, thus simulating conditions upon intravenous nanoemulsion administration.

In addition to the MRC5 cell line, the effectiveness of the formulations was assessed on colon carcinoma cell line (LS). It was found that the curcumin-loaded formulations had an impact on cell viability, while their counterpart placebo prepared in the same dilution had not (Figure 7b). Higher impact on cell viability coming from curcumin itself, compared to the formulations could be explained by the encapsulation of curcumin into the droplets and strong steric protection, which could slow down the release and cellular uptake of curcumin, giving rise to lower activity [49]. Such conclusion is also in line with the in vitro release study.

### 2.7. A Pharmacokinetic Pilot Study

Inherent physicochemical instability of curcumin makes any computational predictions of its bioavailability very unreliable [9]. This is further complicated when investigating the advanced drug delivery systems, such as PEGylated nanoemulsions. In vivo fate of curcumin had been followed for only 60 min [50] when formulated as nanosuspensions, or up to 24 h, when incorporated into zein-polysulfobetaine conjugate-based nanocarriers [51]. This is why the pilot study of pharmacokinetics was warranted—in order to elucidate whether or not there is a difference in curcumin concentration in different tissues and organs 20 min or 24 h after the treatment administration and to further help establishing the time intervals for curcumin detection in selected tissues and organs. It could be noticed that the concentration of curcumin in plasma 20 min after administration was three-fold higher for CP21 and CP51 compared to the curcumin solution and almost two-fold higher compared to CNPEG (Table 8). This could be ascribed to the presence of PEGylated phospholipids in CP21 and CP51 which provide protection against mononuclear phagocytic system (MPS) and allow prolonged circulation of the nanoemulsion droplets [14,52]. On the other hand, 24 h upon administration no significant changes in curcumin plasma concentrations were found between the formulations, probably due to distribution to the extravascular tissues and organs (Table 8). Distribution of curcumin into the brain was studied because it makes for a possible target site for its action [53]. It could be noted that the concentration of curcumin did not differ both 20 min and 24 h upon application and between the formulations (Table 8). This could potentially be explained by the Cmax concentration being reached sometime between these two time points, so it could not be detected at the chosen time points. When it comes to kidneys, it could be observed that after 24 h the concentration was at least two times lower compared to the values obtained after 20 min (Table 8), indicating that curcumin had been eliminated from the kidneys sometime between, probably after metabolization through reduction and conjugation [2,54]. This study shows that, while the nanoemulsion itself, as well as its PEGylated forms prolong circulation time of curcumin, it is almost completely eliminated from all the investigated matrices. Therefore, even though PEGylated NEs seem to be promising carriers for curcumin delivery, a full scale study should be performed, with final time point set at under 24 h, in order to gain a more complete insight into the formulation’s impact on in vivo fate of curcumin upon application.

## 3. Materials and Methods

### 3.1. Materials

Curcumin ((E,E)-1,7-bis(4-Hydroxy-3-methoxyphenyl)-1,6-heptadiene-3,5-dione), polysorbate 80 (polyoxyethylensorbitanmonooleate), benzyl alcohol (BA), butylhydroxytoluene (BHT), α-tocopherol, DPPH (2-Diphenyl-1-(2,4,6-trinitrophenyl)hydrazyl), TPTZ (2,4,6-Tri(2-pyridyl)-s-triazine), iron(II) sulfate heptahydrate and hydrochloric acid were obtained from Sigma-Aldrich Co (St. Louis, MO, USA). Medium chain triglycerides (MCT) were purchased from Fagron GmbH and KG (Barsbüttel, Germany). Soybean oil (Lipoid Purified Soybean Oil 700), soybean lecithin (Lipoid S 75), sodium oleate (Lipoid Sodium Oleate B), PEGylated phospholipids (PEG-PLs)—PEG2000-DSPE (N-(Carbonyl-methoxypolyethylenglycol-2000)-1,2-distearoyl-sn-glycero-3-phosphoethanolamine, sodium salt), and PEG-5000-DPPE (N-(Carbonyl-methoxypolyethylenglycol-5000)-1,2-dipalmitoyl-sn-glycero-3-phosphoethanolamine, sodium salt) were very kindly gifted by Lipoid GmbH (Ludwigshafen, Germany), while glycerol was provided by Merck KGaA (Darmstandt, Germany) and iron(III) chloride from Carl Roth GmbH, Germany. Water used for the preparation of formulations as well as for analyses was ultra-pure, obtained via a GenPure apparatus (TKA Wasseranfbereitungssysteme GmbH, Neiderelbert, Germany). All other chemicals and reagents were of pharmaceutical or HPLC grade and were used without further purification.

### 3.2. Solubility Studies

Shake flask method was used to determine solubility of curcumin in soybean oil, MCT–soybean oil mixtures at 1:1 and 4:1 (*w*/*w*) ratio and containing 2% (*w*/*w*) of soybean lecithin. Curcumin was added in excess to 5 g of the potential oil phase constituents. The mixtures were left to stir at 250 rpm on orbital shaker (IKA^®^ KS 260 basic, IKA^®^ Werke GmbH & Co. KG, Staufen im Breisgau, Germany) for 24 h at 25 ± 2 °C, shielded from light. Subsequently, the mixtures were centrifuged (Centrifuge MPW-56, MPW Med. Instruments, Warsaw, Poland) for 30 min at 5000 rpm and the concentration of curcumin was determined by diluting the supernatant aliquots in methanol and measuring the absorbance of the resulting solutions at 425 nm on spectrophotometer (Evolution 300, Thermo Fisher Scientific, Cambridge, UK).

### 3.3. Nanoemulsion Preparation

Placebo NE (NPEG) was prepared using the hot high-pressure homogenization (HPH) method. Briefly, oil phase (20%, *w*/*w*) was prepared by mixing the ingredients (soybean oil and MCT in 1:4 *w*/*w* ratio, 2% (*w*/*w*) of lecithin, 0.05% (*w*/*w*) of BHT (antioxidant) and 3% (*w*/*w*) of BA) on magnetic stirrer at 50 °C until lecithin was fully dissolved. Aqueous phase was prepared by dissolving polysorbate 80 (2%, *w*/*w*, hydrophilic emulsifier), sodium oleate (0.03%, *w*/*w*, co stabilizer) and glycerol (2.25%, *w*/*w*, for tonicity adjustments) in highly purified water and mixing all the components at the magnetic stirrer at 50 °C. Hydrochloric acid (0.1 M solution) was used to adjust the pH of the aqueous phase below 7. PEGylated formulations for the experimental design (P21, P23; P51, P53) were prepared by the addition of 0.1%/0.3% PEG2000-DSPE to the oil phase or 0.1%/0.3% PEG5000-DPPE to the aqueous phase.

Aqueous phase was added to the oil phase and mixed at 11000 rpm for 1 min using rotor-stator homogenizer (IKA Ultra-Turrax^®^ T25 digital, IKA^®^-Werke GmbH and Co. KG, Staufen, Germany). Coarse emulsion was then further processed by high-pressure homogenizer (EmulsiFlex-C3, Avestin Inc., Ottawa, ON, Canada) for 5/10/15 discontinuous cycles at 500 bar/800 bar. When curcumin-loaded NEs were prepared, curcumin (0.075%, *w*/*w* in the final NE) was dissolved in benzyl alcohol and obtained solution was added to the rest of the oil phase before merging with the aqueous phase.

Optimal formulation factors (PEG-PL type and concentration) and HPH process parameters (number of cycles and homogenization pressure) for curcumin-loaded formulations were determined via experimental design approach. The rest of the preparation process was kept the same as with the placebo formulations. Bearing in mind that curcumin is sensitive to light, all curcumin-loaded formulations were prepared applying shielded protection during oil phase preparation as well as homogenization, both at rotor-stator homogenizer and during HPH. Complete composition of the unloaded (placebo) and curcumin-loaded formulations is given in Table 3.

### 3.4. Experimental Design

The methodology of experimental design was employed to help with the selection of appropriate conditions for the preparation of PEGylated NEs, more precisely, to reveal which combination of formulation factors and process parameters may yield the best PEGylated NE template for curcumin-loaded formulations. With this aim, D-optimal factorial design was applied to identify significant formulation and process variables and their interactions affecting the critical physicochemical attributes of designed NEs, namely droplet size and size distribution, as recognized as critical NEs features in terms of shelf life stability, in vivo fate and potential adverse effects (fat embolism due to the presence of bigger droplets) [15,25,31,32,55]. In view of these considerations, four independent variables chosen for experimental design analysis and their levels were PEG-PL type [A]—PEG2000-DSPE/PEG5000-DPPE; PEG-PL concentration [B]—0.1%/0.3%; HPH pressure [C]—500 bar/800 bar; the number of HPH cycles [D]—5/10/15, while the droplet size (Z-Ave) and polydispersity index (PDI) were predicted as the enough responsive variables. Two process factors—homogenization pressure and number of homogenization cycles—were selected based on the well-known fact that they might affect the NE droplet size and droplet size distribution significantly [31,56,57], whereas two formulation factors—type and concentration of PEG-PL—were set based on the literature overview [14,58,59]. The full scheme of D-optimal design matrix is given in Table 1.

Results obtained for each response were statistically evaluated using Design-Expert^®^ software v. 9.0.1 trial (Stat-Ease Inc., Minneapolis, MN, USA). The first-order polynomial model was applied to fit the experimental data, based on the significant model terms (*p* < 0.05), non-significant lack of fit, multiple correlation coefficient (R^2^), adjusted multiple correlation coefficient (adjusted R^2^) and adequate precision value (a measure of signal to noise ratio, with a desirable value above 4, indicating the model could be used to navigate design space) provided by Design-Expert^®^. Further, the numerical optimization was conducted in order to designate formulations with the highest desirability value, i.e., combinations of input parameters which yield NEs with the smallest Z-ave and PDI. Plots describing interactions between the factors as well as three-dimensional response surface plots displaying evaluated responses (Z-Ave and PDI) at different factor level combinations were constructed.

### 3.5. Sterilization

Given the fact that curcumin is prone to degradation at above 70 °C [60,61] and heat sterilization methods were not applicable, aseptic filtration was used and formulations were filtered through sterile 0.20 µm membrane cellulose acetate filters (Rotilabo, Carl Roth GmbH + Co. KG, Karlsruhe, Germany).

### 3.6. Physicochemical Characterization

#### 3.6.1. Analysis of Droplet Size and Size Distribution

The NE droplet size was determined by dynamic light scattering (DLS), using ZetasizerNano ZS90 (Malvern Instruments Ltd., Worcestershire, UK) and expressed as the mean droplet size (intensity weighted mean diameter, Z-average diameter, Z-Ave) and droplet size distribution (PDI). For the measurements, nanoemulsion samples were diluted with ultra-pure water in 1:500 (*v*/*v*) ratio. The measurements were performed at 25 °C at a fixed scattering angle of 90° using a He-Ne laser at 633 nm.

In order to detect a presence of larger emulsion droplets, laser diffractometry (LD) was used as a complementary method to DLS for droplet sizing. LD measurements were performed applying Universal Liquid Module (ULM) of Beckman Coulter LS 13 320 (Beckman Coulter Inc., Fullerton, CA, USA). Optical model Intralopid.rf780d was used together with PIDSTM (Polarization Intensity Differential Scattering) data in order to obtain information regarding droplet size in the 0.04–2000 µm range. The pump speed and PIDS obscuration were set to 50%. Volume weighted diameters d10, d50, d90 and d100 were used to describe nanoemulsion droplet size distribution.

#### 3.6.2. Analysis of Zeta Potential

Surface charge of nanoemulsion droplets, expressed as zeta potential (ZP), was determined using ZetasizerNano ZS90 (Malvern Instruments Ltd., Worcestershire, UK) by measuring droplets’ electrophoretic mobility and converting it into ZP via software. Samples for the analysis were prepared by diluting nanoemulsions in ultra-pure water, with conductivity value of 50 µS/cm set by addition of 0.9% solution of NaCl, in 1:500 (*v*/*v*) ratio. Measurements were performed at 25 °C.

#### 3.6.3. pH Measurements

Nanoemulsions’ pH values were measured using HI9321 pH meter (Hanna Instruments Inc., Ann Arbor, MI, USA) by direct immersion of the pH electrode into the samples. The measurements were performed at 25 °C.

#### 3.6.4. Conductivity Measurements

Samples’ electrical conductivity was determined using sensION^TM^ + EC71 conductivity meter (ShangHaiShilu Instruments Co., Ltd., Shanghai, China) by directly immersing the electrode in nanoemulsions at 25 °C.

#### 3.6.5. Viscosity Measurements

Rheological analysis was performed by employing MCR 302 air-bearing rheometer (Anton Paar, Graz, Austria) equipped with coaxial cylinders system (CC27 measuring bob in combination with C-PTD 180/Air). Nanoemulsion samples were analyzed initially (48 h after nanoemulsion preparation), and then after two years of storage at room temperature. Viscosity curves and flow curves (continuous rotational tests) were acquired using shear rate range of 0.1–100 s^−1^, at 20 °C. RheoCompasTM software (Anton Paar, Austria) driving the MCR 302 rheometer was programmed to interpolate values of apparent viscosity at shear rate of 50 s^−1^.

#### 3.6.6. Osmolality Testing

The osmolality of samples was determined by using the single sample freezing point osmometer The Advanced^®^ Model 3320 Micro-Osmometer (Advanced Instruments, Inc., Norwood, MA, USA). The nanoemulsion sample (20 µL) was collected into Ease-Eject™ Sampler, placed into the instrument cradle and inserted into the freezing chamber of osmometer.

### 3.7. Microscopical Analysis

#### 3.7.1. Atomic Force Microscopy

A drop (10 µL) of sample—nanoemulsion diluted in ultra-pure water in 1:1000, *v*/*v* ratio—was deposited onto circular mica plate (Highest Grade V1 AFM Mica Discs, Ted Pella Inc., Redding, CA, USA) and dried under vacuum in order to remove the excess of water. Detailed investigation of the morphology of the samples, as well as of the shape, distribution and size of the droplets was accomplished with NTEGRA prima atomic force microscope (NT-MDT). Due to the fragility of the thin layers of the AFM samples, intermittent-contact AFM mode was implemented. For this purpose, NT-MDT NSGO1 rectangular silicon cantilevers with Au reflective film were used. Nominal resonant frequency of these cantilevers is 150 kHz, while nominal force constant is 5.1 N/m. Image Analysis 2.2.0 (NT-MDT) software was implemented for processing of the obtained AFM data.

#### 3.7.2. Optical (Polarization) Microscopy

Optical (polarization) microscopy was employed for the screening of C-PEG-NEs after two-year storage, in order to provide additional information regarding the existence of larger droplets or curcumin crystals. All samples were investigated undiluted, at 1000× magnification using Motic digital DMB3-22ASC microscope (Motic GmbH, Wetzlar, Germany), with or without polarized light lens, equipped with Motic Images Plus v.2.0 software.

### 3.8. Curcumin Assay

The content of curcumin in the prepared nanoemulsion was performed in triplicate by dissolving 50 µL of curcumin loaded nanoemulsion in methanol in 10 mL volumetric flask. The analysis of curcumin content was conducted via LC-MS/MS, using a method previously described by Nikolic et al. [23]. Liquid chromatographic system with Accela autosampler and the pump (Thermo Fisher Scientific, San Jose, CA, USA) was applied, and analysis was conducted under established separation conditions. Xterra^®^ MS C18 column (3.5 μm 2.1 × 150 mm; Waters Corporation, Dublin, Ireland) was used for the separation, at 25 °C. A mobile phase consisting of acetonitrile and 0.1% formic acid aqueous solution (70:30 *v*/*v*) was used for isocratic elution, at flow rate of 0.3 mL/min, and run time of 5 min. Mass analyses were conducted on TSQ Quantum Access MAX triple quadrupole mass analyzer equipped with electrospray ionization (ESI) source and high purity nitrogen as nebulizing gas. Selected reaction monitoring (SRM) for data collecting was in positive mode, and ESI source and mass spectrometry parameters were at following values: spray voltage—5000 V; vaporizer temperature—400 °C; sheath gas pressure—30 units; ion sweep gas pressure—0 units; auxiliary gas—15 units; ion transfer capillary temperature—250 °C; capillary offset—35 units; skimmer offset—0 units; m/z 369.2→177.0; collision energy—22 V; peak width—0.7; scan time—200 ms. The data were processed through Xcalibur software v 2.1.0.1139 (Thermo Fisher Scientific).

### 3.9. Antioxidant Activity

Antioxidant activity of the developed formulations was assessed through two complementary antioxidant assays: DPPH (2,2-diphenyl-1-picrylhydrazyl) assay, which is based on scavenging activity of free-radical and FRAP (ferric ion reducing antioxidant power).

#### 3.9.1. DPPH Assay

Free scavenging activity of curcumin and other more commonly used antioxidants (tocopherol and buthylhidroxitoluen, BHT) was ascertained by preparing different dilutions in isopropanol (0.0188, 0.0375, 0.0563, 0.0750, 0.1125 and 0.1313 mg/mL). Tocopherol was chosen because it bears structural resemblance to curcumin, while BHT is an antioxidant added into the formulation to prevent oil and lecithin oxidation and it was of interest to elucidate its contribution to the antioxidant potential of the formulations from that of curcumin itself. The test was performed by adding 400 μL of each dilution into 3.6 mL of DPPH free radical isopropanol solution (0.1 mM), resulting in final antioxidant concentrations—0.00188, 0.00375, 0.00563, 0.00750, 0.01125 and 0.01313 mg/mL, covering absorbance values between approximately 0.3 and 0.8. Each concentration was prepared in triplicate. The absorbance was recorded at 517 nm using UV/VIS spectrophotometer (Evolution 300, Thermo Fisher Scientific, Cambridge, UK), after 30 min incubation, in the dark, at room temperature. The control sample was prepared by using 400 μL of isopropanol instead of antioxidant dilutions. The free scavenging potential was expressed as inhibition percentage and calculated using the equation I = [(Ac − As)/Ac] × 100; where I stands for inhibition percentage, Ac for the absorbance of the control sample and As for the absorbance of the test sample. The calculated inhibition percentage was plotted against sample concentration and IC50 values (concentration that causes 50% of inhibition or scavenging activity), were determined by linear regression analysis.

Free scavenging activity of selected nanoemulsion formulations was tested with them firstly diluted with highly purified water in order to obtain the same concentration of curcumin as in the isopropanolic solution, and then treated as described in the previous paragraph. In order to establish the preservation of free scavenging activity of curcumin in nanoemulsions, the same assay was applied on samples after two years of storage at room temperature and the IC50 values were compared.

#### 3.9.2. FRAP Assay

In short, 100 μL of nanoemulsion was diluted in water and mixed with 3 mL of FRAP reagent (25 mL of 300 mM acetate buffer (pH 3.6); 2.5 mL of 10 mM TPTZ (2,4,6-tripyridyl-s-triazine) solution in 40 mM HCl; 2.5 mL of 20 mM FeCl_3_ × 6 H_2_O solution in purified water), freshly prepared. The mixture was allowed to incubate in the dark, at controlled temperature of 37 °C, for 30 min. The absorbance was then measured at 593 nm via UV/VIS spectrophotometer (Evolution 300, Thermo Fisher Scientific, Cambridge, UK). The blanks were prepared in the same manner, but instead of curcumin-loaded formulations, dilutions of corresponding placebo nanoemulsions were used. The same test was performed with nanoemulsions after two years of storage as a way of assessing their long-term stability. For comparison, the same test was performed with curcumin, tocopherol and BHT, after dissolving them in methanol. The blank for these samples was prepared by mixing 100 μL of methanol and 3 mL of FRAP reagent. Antioxidant potential (FRAP value) was calculated based on the calibration curve obtained by measuring the absorbance of mixtures made of 100 μL of FeSO_4_ × 7 H_2_O standard solutions (concentrations ranging from 25 to 1200 μmol/L) and 3 mL of FRAP reagent and expressed as mmol Fe^2+^/g of dry matter. The tested concentration of curcumin, BHT, tocopherol (0.0375 mg/mL) was chosen based on the absorbance intensity.

### 3.10. Stability Study

Physicochemical stability of the prepared curcumin-loaded nanoemulsions and corresponding unloaded formulations was monitored during two years of storage at room temperature by measuring aforementioned parameters (Z-ave, PDI, ZP, pH value, conductivity, viscosity and content of curcumin).

### 3.11. In Vitro Release Study

The release of curcumin from the prepared nanoemulsions and curcumin solution CSol taken as reference (0.75 mg/mL in ethanol, 96%, *v*/*v*), was studied via reverse dialysis bag technique, using D-9527 Sigma cellulose membrane dialysis tubing (molecular weight cut-off 12,000). The tubing was soaked overnight in dissolution medium—a mixture of highly purified water and ethanol (1:1, *v*/*v*) chosen in order to provide adequate solubilization of curcumin and prevent its degradation [23,62]. The dialysis tubing/bags were filled with 5 mL of dissolution medium and sealed with dialysis tubing closers. The tubing was placed in a flask containing 200 mL of dissolution medium and covered with aluminum foil in order to prevent the evaporation of ethanol and avoid the light degradation of curcumin. The flasks were equilibrated in ES-20 orbital shaker–incubator (Biosan SIA., Riga, Latvia) at 37 °C. After equilibration, 2 mL of the nanoemulsion sample was added into the flask (donor chamber), and stirred at 100 rpm, maintaining the temperature at 37 °C. After 5, 10, 30, 60, 90 and 180 min the dialysis bag (acceptor phase) was removed from the flask and analyzed for curcumin content by above mentioned LC-MS/MS (please see Section 3.8. Curcumin assay). The analysis was performed in triplicate. The release pattern of curcumin from selected formulations was calculated by plotting cumulative percentage drug release versus time. The release kinetics of curcumin from the investigated NEs was evaluated by fitting the experimentally obtained release data through several mathematical models (zero-order, first-order, Higuchi, Baker–Lonsdale, Korsmeyer–Peppas and Hixson–Crowell), by employing DDSolver add-in program for the Microsoft Excel application.

### 3.12. Hemolysis Testing

Hemolytic potential of selected nanoemulsions was assessed via modified method previously described by Amin et al. and Devalapally et al. [48,59]. Briefly, two identical sets of samples containing 0.1 mL of nanoemulsion and 0.9 mL of citrated whole rat blood were vortexed for 2–3 s and equilibrated at 25 °C for 2 min. Two controls, saline (S) and 2% sodium dodecyl sulfate solution (SDS) were used as 0% and 100% of hemolysis, respectively. After the end of incubation, 5 mL of saline was added to the samples, they were briefly vortexed and centrifuged for 5 min at 3000 rpm (Hermle Z 206 A, Wehingen, Germany). This step was repeated for four times, discarding supernatants. Finally, 4 mL of highly purified water was added into the remaining plug, vortexed and centrifuged for 5 min at 3000 rpm to release the remaining hemoglobin. Resulting supernatant was collected and the absorbance was measured at 540 nm, proportionally to the amount of intact red blood cells. Hemolytic potential of nanoemulsions was expressed as % hemolysis and calculated via equation: % hemolysis = [(ASal − As)/(ASal − ASDS)] × 100; where ASal is absorbance value for samples containing saline; AS is absorbance for nanoemulsion samples and ASDS is absorbance for samples with SDS. Formulations with hemolytic potential values were under 10% are deemed nonhemolytic, while values over 25% indicate a hemolytic formulation [48].

### 3.13. Cytotoxic Activity

#### 3.13.1. Cell Culture

Human colon carcinoma cells (LS-174) and normal human lung fibroblasts cells (MRC5), kindly gifted from the Institute of Oncology and Radiology of Serbia, were maintained as monolayer culture in the Roswell Park Memorial Institute (RPMI) 1640 nutrient medium (Sigma Chemicals Co., St. Louis, MO, USA). RPMI 1640 nutrient medium was prepared in sterile deionized water, supplemented with penicillin (100 U/mL), streptomycin (100 mg/mL), 4-(2-hydroxyethyl) piperazine-1-ethanesulfonic acid (HEPES) (25 mM), L-glutamine (3 mM) and 10% of heat-inactivated fetal calf serum (FCS) (pH 7.2). The cells were grown at 37 °C in 5% CO_2_ and humidified air atmosphere.

#### 3.13.2. Cytotoxicity Assay

Cells were seeded in 96-well cell culture plates (Nunc)—LS-174 (7000 *c*/*w*) and MRC5 (5000 *c*/*w*) in culture medium, in total volume of 100 μL, and grown for 24 h. The following day, after the adherence, cells were treated with prepared dilutions of selected nanoemulsions and curcumin. Briefly, stock dispersions of curcumin-loaded nanoemulsions and corresponding placebo formulations were filtered through non-pyrogenic sterile filter 0.22 µm (Sarstedt, Germany) and diluted with nutrient medium (1:4, *v*/*v*). Additionally, curcumin solution was prepared by dissolving curcumin in DMSO, and diluting it with the same nutrient medium in order to obtain the same concentration of curcumin as in the diluted nanoemulsion formulations. Concentration of DMSO in the cell did not exceed 1%. All preparations (nanoemulsion and curcumin dispersions) were further diluted in nutrient medium and 50 µL of the obtained working solutions was added to the wells. Final concentrations of curcumin tested on cancer cell lines were 3.125, 6.25, 12.5 and 25 µg/mL. Dispersions of various concentrations were added to the wells, except the control wells where only nutrient medium was added. All tests were carried out in triplicate. Nutrient medium with corresponding agent concentrations, but without target cells was used as blank, also in triplicate.

Cell survival was assayed 48 h after the treatment, using MTT test. Briefly, 20 μL of 3-(4,5-dimethylthiazol-2-yl)-2,5-diphenyl tetrazolium bromide (MTT, Sigma-Aldrich, Hamburg, Germany) solution (5 μg L-1 in phosphate buffered saline; pH 7.2) was added to each well. Samples were then incubated for four hours at 37 °C in 5% CO_2_ and humidified air atmosphere. Afterwards, 100 μL of 10% sodium dodecyl sulfate was added to the wells and the plates were kept overnight in the CO_2_ incubator in humidified atmosphere, followed by absorbance measurements. The absorbance (A) was measured at 570 nm. The cell survival (%) was calculated by division of A of sample with cells grown in the presence of various concentrations of investigated compounds, with control absorbance (Ac) of cells grown only in nutrient medium and multiplied with 100. It was implied that A of blank was always subtracted from A of corresponding sample with target cells.

### 3.14. Pharmacokinetic Study—A Pilot Trial on Rats

Pharmacokinetic studies were conducted after intravenous administration of nanoemulsions, or curcumin solution (0.075% curcumin, 5% DMSO, 5% PEG 400, 2% polysorbate 80, 14% propylene glycol and highly purified water to 100%). Male Sprague-Dawly rats, 8–10 weeks old and weighing 150–200 g were housed four animals per cage on a 12 h light/dark period (light on at 6:00 a.m). In the animal room the temperature was maintained at 22 ± 1 °C, with the relative humidity 40–70%, and the illumination 120 lx. The study was conducted according to the National Institutes of Health Animal Care and Use Committee guidelines and was approved by the Ethical Committee on Animal Experimentation of the University of Belgrade—Faculty of Pharmacy, Serbia, document number 323-07-13806/2020-05 from 31 December 2020.

Experimental animals were divided into four groups based on the received treatment, each group counting four animals (two animals were used per time point for each formulation). Formulations were administered intravenously through tail vein using syringe pump (Stoelting Co., Wood Dale, IL, USA) in 6.67 mL/kg volume, in order to achieve a dose of 5 mg/kg. Five minutes or 24 h after administration animals were anesthetized by ketamine hydrochloride (90 mg/kg, 10% Ketamidor, Richter Pharma AG, Wels, Austria), and samples of blood (collected via cardiac puncture in heparinized syringes), liver, kidneys and brain were collected. Plasma was obtained after 10 min of centrifugation at 1000× *g* (MiniSpin^®^ plus centrifuge, Eppendorf, Germany). Tissue samples were weighed and homogenized in 80% (*v*/*v*) acetonitrile aqueous solution in 1:3 (*w*/*v*, g/mL) ratio with IKA Ultra-Turrax^®^ (T25 digital, IKA^®^-Werke GmbH and Co. KG, Staufen, Germany) for 1 min at around 16,000 rpm. Supernatants were isolated after centrifugation for 20 min at 4000 rpm (Hermle Z 206 A, Wehingen, Germany). Plasma and tissue supernatants (170 µL) were further purified by protein precipitation method, using 415 µL of acetonitrile with the addition of 15 µL of internal standard solution. Calibration samples were prepared by mixing of 30 µL of the standard curcumin methanolic solutions (0.5, 1, 2, 2.5, 3, 3.5 µM for plasma, kidney and brain samples and 0,25, 0,5, 1, 2, 3, 3,5 µM for the liver samples) using corresponding biological material from untreated animals (170 µL), 15 µL of internal standard solution and 385 µL of acetonitrile. These samples were further vortexed for 60 s, and centrifuged at 4 °C for 10 min at 12,000× *g* (Eppendorf 5424R centrifuge, Eppendorf AG, Hamburg, Germany), and supernatants were analyzed by LC-MS/MS.

### 3.15. Statistical Analysis

Statistical analysis was performed with the help of IBM SPSS Statistics software (v.23) (Chicago, IL, USA) Whenever possible, results were presented as mean values of observed parameter ± SD, with the exception of the pharmacokinetic study where the results were presented as mean ± S.E.M. Statistical analysis of differences in measured physicochemical parameters: upon preparation and after two years of storage (for curcumin-loaded NEs), between CNPEG and C-PEG-NEs, between placebo and corresponding curcumin-loaded NEs as well as between FRAP values was performed using Student’s *t*-test. For the analysis of the results obtained from in vitro release testing, one-way ANOVA, followed by Tukey HSD post hoc test was used. When the assumptions of ANOVA were not met (the variances of the groups were not equal), a nonparametric Kruskal–Wallis test (one-way ANOVA by ranks) was performed together with the nonparametric Mann–Whitney *U* test, for pairwise group comparisons, in the case of significant difference. The *p* value of 0.05 was taken as the level of significance.

## 4. Conclusions

In the current study, experimental design strategy was successfully applied to obtain the best template for long-term stable curcumin-loaded nanoemulsion formulations, intended for parenteral use. The results of the D-optimal factorial design indicated that not only investigated factors alone, but also their interactions could significantly affect nanoemulsion characteristics. Based on numerical optimization data, both investigated PEG-PLs (PEG2000-DSPE, PEG5000-DPPE), in both proposed concentrations (0.1%, 0.3%), combined with HPH pressure of 800 bar and 10 homogenization cycles (chosen as optimal process parameters), were suggested to provide nanoemulsions with satisfactory quality attributes for parenteral application and were selected as templates for curcumin solubilisation. Formulated curcumin-loaded NEs revealed small mean droplet size (Z-ave: 104.7 ± 2.7 nm), narrow size distribution (PDI: 0.073 ± 0.019), high surface charge (ZP: −41 ± 4.8 mV), low viscosity (30.471 ± 8.801 mPa*s), and acceptable osmolality (549 ± 12 mOsm/kg), thus implying their suitability as carriers for intravenous administration of poorly water-soluble drugs. AFM analysis showed the absence of curcumin crystals, and corroborated size measurements for PEGylated NE formulations. After two years of storage at room temperature, all formulations remained stable in terms of their physicochemical characteristics and curcumin content. In vitro release studies proved that PEGylated phospholipids slow down the release of curcumin, which was more pronounced in formulations containing PEG2000-DSPE, but both formulations containing 0.1% of PEG-PLs (CP21 and CP51) decreased the release of curcumin more in comparison with the formulations containing 0.3% of PEG-PLs (CP23 and CP53), which were, therefore, chosen for further research. Antioxidant assays further supported the claim that developed nanoemulsions were successful in maintaining the activity of curcumin. The safety of the formulations was shown as satisfactory in hemolytic and cytotoxicity assays., Pilot pharmacokinetic study showed that the concentration of curcumin in plasma was the highest after the administration of PEGylated, CP21 curcumin-loaded nanoemulsion, but a full scale pharmacokinetic study is needed to provide a more detailed insight into the impact of PEGylated phospholipids on bioavailability of curcumin, with the CP21 and CP51 standing out as promising candidates for improved curcumin delivery.

## Figures and Tables

**Table 1:** D-optimal experimental design matrix and the measured values for the responses.

	Factors								Responses	
	A: PEG-PL Type		B: PEG-PL Concentration		C: HPH Pressure		D: HPH Cycles		Z-Ave	PDI
	AL	CL	AL	CL	AL	CL	AL	CL		
1 *	PEG2000-DSPE	−1	0.10%	−1	500 bar	−1	5	{−1 1}	160.3 ± 2.2	0.158 ± 0.009
2							10	{0–2}	132.6 ± 2.0	0.161 ± 0.015
3					800 bar	1	10	{0–2}	108.8 ± 0.6	0.125 ± 0.022
4 *							15	{1 1}	106.1 ± 1.0	0.117 ± 0.005
5			0.30%	1	500 bar	−1	5	{−1 1}	169.0 ± 2.9	0.171 ± 0.010
6							10	{0–2}	128.8 ± 1.9	0.148 ± 0.003
7							15	{1 1}	114.5 ± 1.2	0.118 ± 0.009
8					800 bar	1	5	{−1 1}	131.2 ± 1.2	0.112 ± 0.035
9							10	{0–2}	111.8 ± 0.6	0.136 ± 0.006
10 *							15	{1 1}	110.4 ± 1.5	0.154 ± 0.021
11	PEG5000-DPPE	1	0.10%	−1	500 bar	−1	5	{−1 1}	165.0 ± 2.4	0.153 ± 0.006
12							10	{0–2}	135.0 ± 2.1	0.163 ± 0.018
13							15	{1 1}	122.8 ± 1.7	0.139 ± 0.021
14					800 bar	1	5	{−1 1}	126.4 ± 0.6	0.101 ± 0.009
15							10	{0–2}	110.2 ± 1.7	0.100 ± 0.017
16							15	{1 1}	108.7 ± 1.3	0.113 ± 0.015
17			0.30%	1	500 bar	−1	5	{−1 1}	171.2 ± 2.3	0.175 ± 0.018
18 *							15	{1 1}	125.6 ± 2.5	0.152 ± 0.029
19					800 bar	1	5	{−1 1}	125.2 ± 1.1	0.161 ± 0.019
20 *							10	{0–2}	108.6 ± 1.5	0.115 ± 0.013

* Replicated runs; AL: actual level; CL: coded level.

**Table 2:** Numerical optimization of the formulations selected via experimental design.

	PEG-PL Type	PEG-PL Concentration (%, *w*/*w*)	HPH Pressure (bar)	HPH Cycle	Desirability Value
1	PEG5000-DPPE	0.1	800	10	0.761
2	PEG2000-DSPE	0.1	800	10	0.666
3	PEG2000-DSPE	0.1	800	15	0.636
4	PEG5000-DPPE	0.1	800	15	0.559
5	PEG5000-DPPE	0.3	800	10	0.556
6	PEG2000-DSPE	0.1	500	15	0.552
7	PEG2000-DSPE	0.3	800	10	0.476
8	PEG2000-DSPE	0.3	800	15	0.385
9	PEG5000-DPPE	0.3	800	15	0.332
10	PEG2000-DSPE	0.3	500	15	0.188

**Table 3:** Composition of placebo and curcumin-loaded nanoemulsions.

	Nanoemulsion Formulations									
Ingredients (%, *w*/*w*)	NPEG	P21	P23	P51	P53	CNPEG	CP21	CP23	CP51	CP53
Oil phase										
Curcumin	-	-	-	-	-	0.075	0.075	0.075	0.075	0.075
Soybean oil	4	4	4	4	4	4	4	4	4	4
MCT	16	16	16	16	16	16	16	16	16	16
LS	2	2	2	2	2	2	2	2	2	2
BA	3	3	3	3	3	3	3	3	3	3
BHT	0.05	0.05	0.05	0.05	0.05	0.05	0.05	0.05	0.05	0.05
PEG2000-DSPE	-	0.1	0.3	-	-	-	0.1	0.3	-	-
Aqueous phase										
P80	2	2	2	2	2	2	2	2	2	2
Glycerol	2.25	2.25	2.25	2.25	2.25	2.25	2.25	2.25	2.25	2.25
SO	0.03	0.03	0.03	0.03	0.03	0.03	0.03	0.03	0.03	0.03
PEG5000-DPPE	-	-	-	0.1	0.3	-	-	-	0.1	0.3
Water to	100	100	100	100	100	100	100	100	100	100

MCT: medium chain triglycerides; BA: benzyl alcohol; BHT: butyl hydroxytoluene; LS: soybean lecithin; P80: polysorbate 80; SO: sodium oleate.

**Table 4:** Values of Z-ave, PDI and ZP for curcumin-loaded and placebo nanoemulsions measured initially and after two years of storage at room temperature.

	Z-Ave (nm)		PDI		ZP (mV)	
	Initial	2 Years	Initial	2 Years	Initial	2 Years
CNPEG	101.4 ± 0.2	135.0 ± 2.7 ^###^	0.076 ± 0.018	0.061 ± 0.020	−48.9 ± 4.2	−41.1 ± 0.4
CP21	103.6 ± 0.3 ***	112.7 ± 1.3 ^###^	0.073 ± 0.012	0.121 ± 0.029	−38.7 ± 0.2	−44.9 ± 1.4 ^##^
CP23	107.7 ± 0.1 ***	112.2 ± 2.3	0.102 ± 0.003	0.148 ± 0.016 ^#^	−41.5 ± 0.3	−43.3 ± 1.7
CP51	103.4 ± 0.1 ***	111.0 ± 2.4 ^##^	0.048 ± 0.008	0.099 ± 0.003 ^###^	−39.6 ± 1.0 *	−37.7 ± 1.0
CP53	107.4 ± 0.6 ***	113.3 ± 2.3 ^#^	0.068 ± 0.017	0.117 ± 0.019 ^#^	−36.3 ± 0.9 **	−40.8 ± 0.8 ^##^
NPEG	102.9 ± 1.2	128.7 ± 1.9	0.055 ± 0.019	0.094 ± 0.014	−46.6 ± 0.6	−45.0 ± 2.2
P21	104.8 ± 1.2	109.7 ± 1.4	0.053 ± 0.015	0.100 ± 0.026	−47.3 ± 1.2 ^+++^	−44.0 ± 0.6
P23	103.5 ±0.4 ^+++^	110.1 ± 0.6	0.066 ± 0.012 ^++^	0.116 ± 0.027	−39.2 ± 1.8	−43.1 ± 1.40
P51	104.5 ± 0.8	114.9 ±0.5	0.053 ± 0.019	0.108 ± 0.022	−37.5 ± 0.8 ^+^	−40.1 ± 0.5
P53	105.3 ± 0.4 ^++^	111.5 ± 1.0	0.074 ± 0.011	0.110 ± 0.013	−34.3 ± 0.9	−38.1 ± 0.5

Values are shown as means ± sd (*n* = 3); *, ** and ***, *p* < 0.05; *p* < 0.01 and *p* < 0.001 compared to CNPEG; ^#^, ^##^ and ^###^, *p* < 0.05; *p* < 0.01 and *p* < 0.001 compared to the initial values for curcumin-loaded nanoemulsions; ^+^, ^++^ and ^+++^, *p* < 0.05; *p* < 0.01 and *p* < 0.001 curcumin-loaded vs. corresponding placebo nanoemulsion.

**Table 5:** Values of pH, conductivity and viscosity for curcumin-loaded and placebo nanoemulsions measured initially and after two years of storage at room temperature.

	pH		Conductivity (µS/cm)		Viscosity (mPa*s)	
	Initial	2 Years	Initial	2 Years	Initial	2 Years
CNPEG	6.84 ± 0.03	5.53 ± 0.01 ^###^	239.33 ± 1.53	175.90 ± 1.21 ^###^	28.476	5.599
CP21	6.89 ± 0.01	5.8 ± 0.03 ^###^	229.33 ± 0.58 ***	164.33 ± 2.47 ^###^	23.405	13.949
CP23	6.86 ± 0.02	5.85 ± 0.02 ^###^	301.00 ± 1.73 ***	212.00 ± 1.73 ^###^	26.432	12.463
CP51	6.76 ± 0.04	6.02 ± 0.01 ^###^	254.67 ± 1.16 ***	176.17 ±0.32 ^###^	28.250	17.391
CP53	6.76 ± 0.02 *	4.78 ± 0.02 ^###^	232.00 ± 1.73 **	198.67 ± 1.60 ^###^	45.790	10.927
NPEG	6.78 ± 0.02	5.49 ± 0.01 ^+^	155.87 ± 2.70 ^+++^	152.43 ± 1.50	n.d.	n.d.
P21	6.89 ± 0.02	5.79 ± 0.01	162.40 ± 0.53 ^+++^	155.73 ± 0.67	n.d.	n.d.
P23	6.85 ± 0.02	5.90 ± 0.01	178.83 ± 0.67 ^+++^	241.00 ± 3.46	n.d.	n.d.
P51	6.77 ± 0.03	5.52 ± 0.03	157.20 ± 0.92 ^+++^	261.33 ± 2.31	n.d.	n.d.
P53	6.70 ± 0.02	5.48 ± 0.03 ^+^	175.70 ± 2.23 ^+++^	164.50 ± 2.10	n.d.	n.d.

Values are shown as means ± sd (*n* = 3); *, ** and ***, *p* < 0.05; *p* < 0.01 and *p* < 0.001 compared to CNPEG; ^###^, *p* < 0.001 compared to the initial values for curcumin-loaded nanoemulsions; ^+^, and ^+++^, *p* < 0.05 and *p* < 0.001 curcumin-loaded vs. corresponding placebo nanoemulsion; n.d. not determined.

**Table 6:** The impact of aseptic filtration on droplet size (Z-ave) and polydispersity index (PDI) of C-PEG-NEs.

	Z-Ave (nm)		PDI	
	Before Filtration	After Filtration	Before Filtration	After Filtration
CNPEG	101.4 ± 0.2	102.1 ± 0.3 *	0.076 ± 0.018	0.072 ± 0.017
CP21	103.6 ± 0.3	104.5 ± 0.3 *	0.073 ± 0.012	0.078 ± 0.011
CP23	107.7 ± 0.1	105.6 ± 0.6 **	0.102 ± 0.003	0.064 ± 0.004 ***
CP51	103.4 ± 0.1	106.4 ± 0.4 ***	0.048 ± 0.008	0.100 ± 0.008 ***
CP53	107.4 ± 0.6	108.3 ± 0.8	0.068 ± 0.017	0.105 ± 0.013 *

Values are shown as means ± sd (*n* = 3); *, ** and ***, *p* < 0.05; *p* < 0.01 and *p* < 0.001 compared to before filtration.

**Table 7:** IC50 values from DPPH assay on selected nanoemulsion formulations. The values are presented as mean ± sd (*n* = 3).

Formulation	IC50 (mg/mL)	
	Initial	2 Years
CNPEG	0.0807 ± 0.0019	0.0748 ± 0.0010
CP21	0.0788 ± 0.0006	0.0750 ± 0.0007
CP51	0.0779 ± 0.0007	0.0725 ± 0.0019

**Table 8:** Concentrations of curcumin in plasma, brain, liver and kidneys after intravenous administration of 5 mg/kg of solution, CNPEG, CP21 and CP51. The results are presented as mean ± SEM (*n* = 2).

	Solution		CNPEG		CP21		CP51	
**Time (h)**	0.33	24	0.33	24	0.33	24	0.33	24
	**Concentration (ng/mL)**							
**Plasma**	129.70 ± 14.78	32.92 ± 1.70	255.39 ± 38.49	30.58 ± 4.31	398.33 ± 9.12	26.88 ± 2.86	339.69 ± 12.60	24.27 ± 0.32
	**Concentration (ng/g)**							
**Brain**	108.07 ± 0.17	109.33 ± 2.37	112.26 ± 0.02	109.46 ± 2.09	109.58 ± 0.92	107.57 ±0.12	107.10 ± 0.47	107.90 ± 1.28
**Liver**	76.44 ± 3.57	74.64 ± 3.69	68.01 ± 1.62	66.23 ± 2.30	84.48 ± 10.53	69.16 ± 1.67	68.79 ± 2.61	70.68 ± 1.45
**Kidneys**	145.67 ± 13.31	43.50 ± 1.90	118.11 ± 42.87	45.62 ± 6.18	200.98 ± 65.90	44.01 ± 4.49	116.59 ± 21.30	44.71 ± 0.18

## Supplementary Materials

The following are available online at https://www.mdpi.com/article/10.3390/ijms22157991/s1.
