# Supplementary material for: Curcumin Loaded PEGylated Nanoemulsions Designed for Maintained Antioxidant Effects and Improved Bioavailability: A Pilot Study on Rats
Source: Int J Mol Sci. 2021 Jul 27;22(15):7991. doi: 10.3390/ijms22157991 (PMC8347926; doi:10.3390/ijms22157991)
Supplement: Supplementary file 1 [file ijms-22-07991-s001.zip › ijms-1293497-sup-proof done.pdf]

Supplementary material

Table S1. Model confirmation – comparison of predicted and observed values for nanoemulsion droplet size and size distribution

Factor settings				Response	Predicted mean	SE Pred	95% PI low	95% PI high	Observed mean ± SD
PEG-PL type	PEG-PL concentration	HPH pressure	HPH cycle						
PEG5000-DPPE	0.1%	800 bar	10	Z-ave (nm)	107.2	1.7	103.5	110.8	107.7 ± 1.1
				PDI	0.105	0.01	0.082	0.127	0.084 ± 0.01
PEG2000-DSPE	0.1%	800 bar	10	Z-ave (nm)	110.2	1.8	106.3	114.1	106.9 ± 0.8
				PDI	0.105	0.01	0.083	0.127	0.085 ± 0.013
PEG5000-DPPE	0.3%	800 bar	10	Z-ave (nm)	108.6	1.6	105.2	112.1	109.4 ± 0.8
				PDI	0.123	0.01	0.101	0.144	0.102 ± 0.009

SE: standard error; PI: predicted interval; SD: standard deviation.

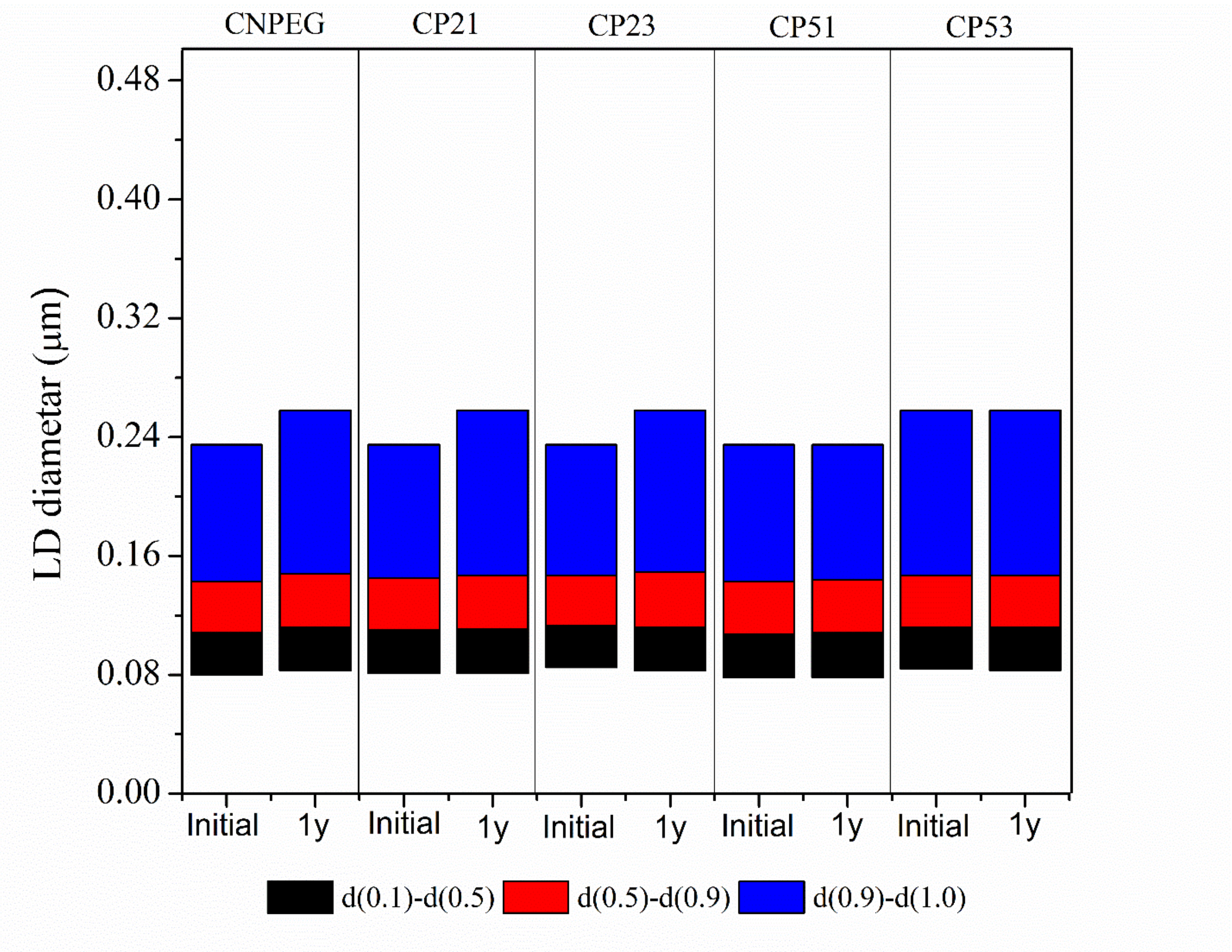

Figure S1. Droplet size of curcumin loaded formulations measured by laser diffraction (LD) initially and after 1 year of storage at room temperature

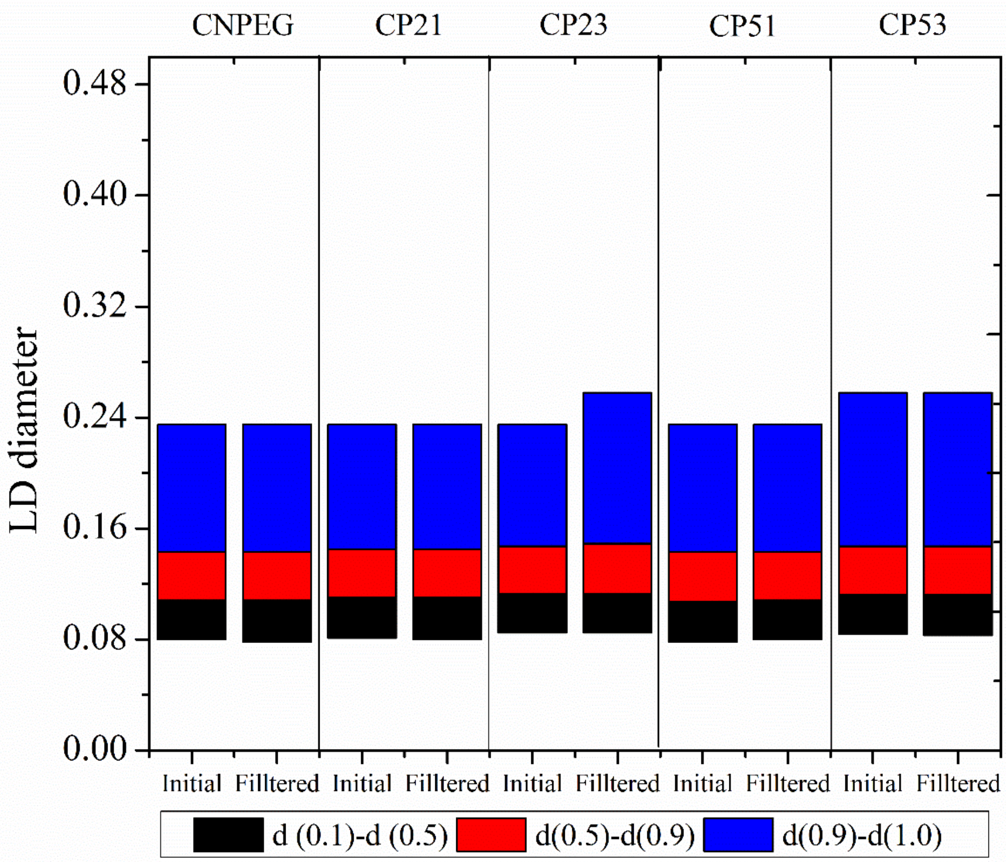

Figure S2. Droplet size of curcumin loaded formulations measured by laser diffraction (LD) initially and after aseptic filtration

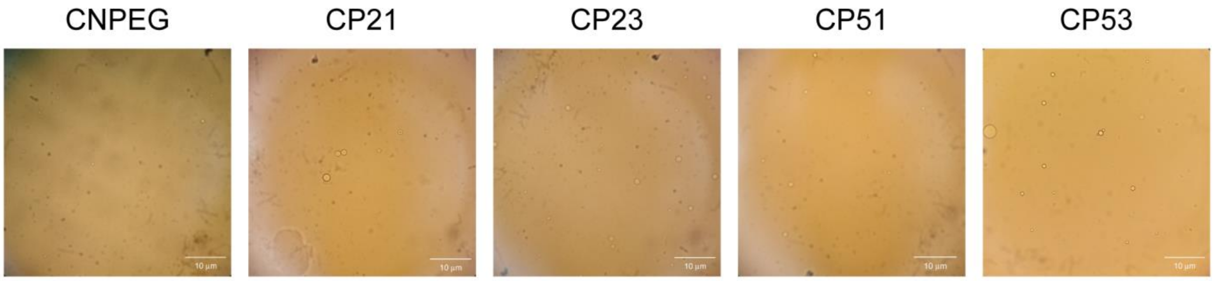

Figure S3. Curcumin-loaded NEs as observed under optical microscope after two years of storage. All micrographs were taken under 1000x magnification

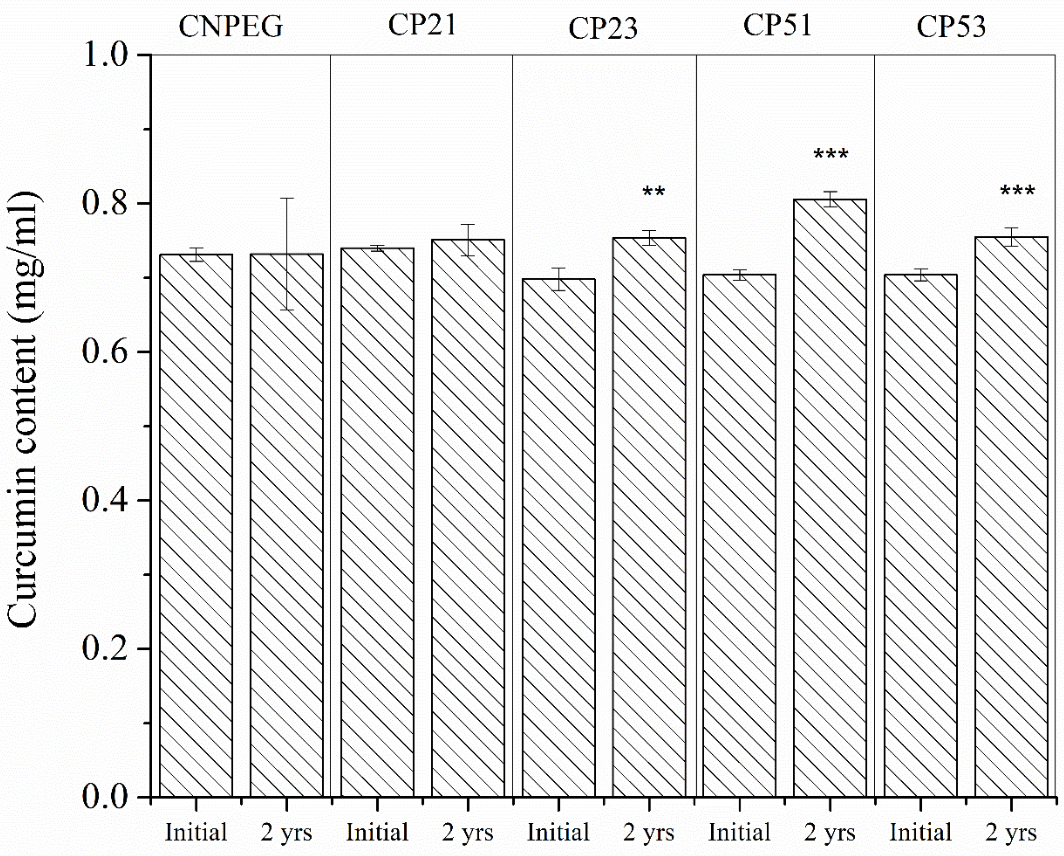

Figure S4. Curcumin content in formulations (mg/ml) measured initially and after two years of storage; Values are shown as means ± sd (n = 3); *, ** and ***, $p<0.05$; $p<0.01$ and $p<0.001$ compared to the initial values

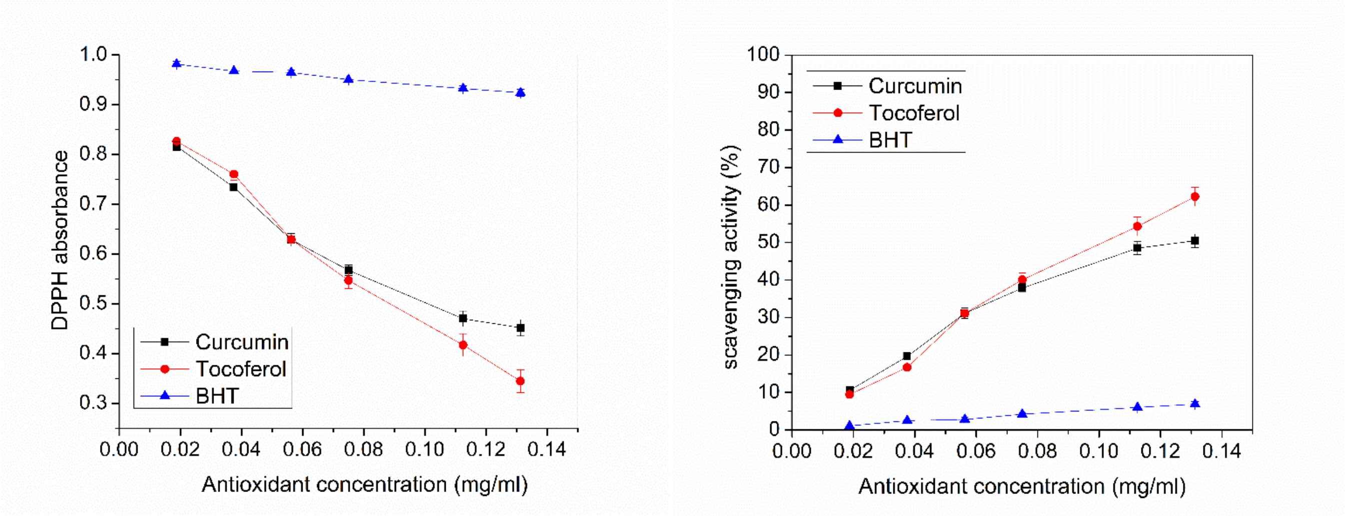

(a) (b)

Figure S5. (a) DPPH absorbance measured after exposure to different antioxidants; (b) corresponding free radical scavenging activity as the function of antioxidant concentration; Values are shown as means ± sd (n = 3)

Table S2. *In vitro* release kinetic data of curcumin from the investigated nanoemulsions

Kinetic model	Parameter	Nanoemulsion formulation				
		**CNPEG**	**CP21**	**CP23**	**CP51**	**CP53**
Zero-order	K	0.2100	**0.1386**	**0.1881**	**0.2064**	**0.2294**
	R^2	0.9784	**0.9388**	**0.9656**	**0.9670**	**0.9711**
	$R^2_{adjusted}$	0.9784	**0.9388**	**0.9656**	**0.9670**	**0.9711**
	AIC	19.8022	**21.7451**	**22.2229**	**22.6885**	**23.9795**
First-order	K	0.0025	0.0016	0.0023	0.0025	0.0029
	R^2	0.9804	0.9263	0.9402	0.9600	0.9378
	$R^2_{adjusted}$	0.9804	0.9263	0.9402	0.9600	0.9378
	AIC	19.4865	22.7199	25.5555	24.2437	28.3969
Higuchi	K	2.2064	1.3987	1.8956	2.1276	2.3119
	R^2	0.8274	0.7047	0.7168	0.7695	0.7210
	$R^2_{adjusted}$	0.8274	0.7047	0.7168	0.7695	0.7210
	AIC	32.5210	31.9964	35.4076	34.7768	37.7045
Baker–Lonsdale	K	0.0002	0.0001	0.0001	0.0002	0.0002
	R^2	0.6206	0.4166	0.3697	0.5346	0.3601
	$R^2_{adjusted}$	0.6206	0.4166	0.3697	0.5346	0.3601
	AIC	37.2116	36.0487	40.1841	38.9333	42.6476
Korsmeyer–Peppas	K	0.0272	0.0392	0.001	0.0337	0.0179
	N	1.4866	1.2867	1.6296	1.3817	1.5398
	R^2	0.4218	0.9148	0.8314	0.9405	0.9686
	$R^2_{adjusted}$	0.2772	0.8935	0.7893	0.8806	0.9607
	AIC	40.8994	26.4862	32.7895	30.1642	23.9326
Hixson Crowel	K	**0.0008**	0.0005	0.0007	0.0008	0.0008
	R^2	**0.9814**	0.9312	0.9502	0.9641	0.9641
	$R^2_{adjusted}$	**0.9814**	0.9312	0.9502	0.9641	0.9641
	AIC	**19.1794**	22.3467	24.4452	23.5250	23.5250

K, release rate constant; R^2, coefficient of determination; R^2adjusted, adjusted coefficient of determination; AIC, Akaike Information Criterion; n, diffusion release exponent ($n \leq 0.43$ – Fick diffusion; $0.43 < n < 0.85$ – anomalous transport, non-Fickian diffusion; $n \geq 0.85$ – zero order release);
